# Processing-Driven Structuring of Polymer-Based Materials: A Brief Overview

**DOI:** 10.3390/polym17182483

**Published:** 2025-09-14

**Authors:** Fulvia Cravero, Rossella Arrigo, Alberto Frache

**Affiliations:** 1 Department of Applied Science and Technology, Politecnico di Torino, Viale Teresa Michel 5, 15121 Alessandria, Italy; fulvia.cravero@polito.it (F.C.); alberto.frache@polito.it (A.F.); 2 Local INSTM Unit, 15121 Alessandria, Italy

**Keywords:** polymer structuring, extrusion, injection molding, polymer blend, hierarchical

## Abstract

Polymer structuring is a valuable cost- and time-saving strategy for the production of high-performance polymer-based materials. The main issue in the spreading of this approach lies in the understanding of the relationships between the processing parameters, the microstructure and the resulting properties, which represent fundamental factors in the actual defining of the final characteristics owing to the production method employed. The aim of the present work is to provide a wide overview of the currently available knowledge on solvent-free approaches for obtaining structured materials, specifically focusing on extrusion- and injection molding-based technologies, given their relevance as the most industrially exploited methods for the melt processing of thermoplastic materials. Additionally, particular attention will be paid to the relationships between the variation in the processing parameters and the resulting flow fields (both shear and elongational), considering their role in the definition of microstructure.

## 1. Introduction

Polymer structuring usually refers to the possibility of obtaining tailored morphologies in polymer-based systems, which are especially desired for the achievement of specific properties. This approach needs deep knowledge of the relationships between the processing parameters and the resulting microstructure, which ultimately determines the final properties of a material [1,2,3,4,5,6,7,8,9,10,11,12,13,14,15,16,17,18,19,20,21,22,23]. Additionally, when more than two levels of morphological organization are identified, a multiscale topology is referred to as a hierarchical microstructure, and the final performance is determined by interactions occurring at molecular, nano, micro and macro scales [3,24,25,26,27]. In particular, hierarchical structures can be obtained in both single-polymer and blend systems. In the first case, a multiscale morphology is achieved by organizing the crystalline phase into multilevel structures, such as the shish kebab, characterized by a cylindrical central skeleton of aligned macromolecules from which epitaxial crystals of oriented lamellae grow at regular intervals [22,28,29,30,31,32,33,34,35,36,37,38,39,40,41,42]. On the other hand, when all-polymer blends are considered, the hierarchical structures are formed due to the mutual organization of the two polymer domains. For instance, this can be achieved by the formation of hybrid shish-kebab structures [25,43,44,45,46,47,48,49,50,51,52,53] or microfibrils [1,20,39,54,55,56,57,58,59,60,61,62,63,64,65,66,67,68,69,70]. The latter are anisotropic micrometer-sized morphologies that can be oriented, and their density can be increased by adjusting the processing parameters. It is interesting to note that all of these were inspired by nature. In fact, tendons, bones, wood, grass, nacre, silk fibers, the pads of geckos, lotus leaves and intervertebral disks are good examples of how a proper microstructure allows the achievement of superior mechanical properties and lightweightness, as well as outstanding adhesion characteristics and self-cleaning properties [3,4,21,23,24,25,71,72,73,74,75,76,77,78].

The ability to engineer the morphology and thus the final properties creates significant opportunities for the application of such structured materials in high-performance technological areas [2,13,14,24], such as adhesive tapes [72], self-cleaning surfaces [71], coating applications and drug delivery [79,80]. On the other hand, hierarchically structured materials are particularly performant in terms of mechanical properties thanks to the efficient transfer of the loads, both in terms of tensile strength and energy adsorption during impact, which is related to the synergistic response of the entire material due to the multiscale design [3,20,22,27,39,81,82,83]. Thus, the interest in promoting the diffusion of hierarchically structured polymer-based materials seems clear. In the case of all-polymer and single-polymer materials, the processes that allow polymer structuring can be divided into two approaches, depending on the presence of a solvent. In particular, when a solvent is present, they can be referred to as solvent-assisted techniques, while alternatively they can be called solvent-free processes [2,5,13,14,20,26,71,84,85,86,87,88,89,90,91,92,93,94].

### 1.1. Solvent-Assisted Production of All-Polymer Materials

In the case of solvent-assisted methods, structuring is mainly achieved by the self-assembly ability of the macromolecules, and microstructure tuning is primarily achieved by controlling the chain structure and the nature of the solvent [26,71,84,85,86,87,88,92,93,94,95,96,97]. Specifically, homopolymers or copolymers can be used. In the case of copolymers, an additional degree of freedom comes from the choice of monomer types and their order within the chains. In fact, the morphology of a two-component multiblock copolymer can be tailored by varying the block lengths and the stiffness of each section. This approach typically results in the formation of core–shell micelles or multilayer structures. In this context, solvent-assisted methods can be viewed as bottom-up design techniques, where the microstructure is shaped by first defining the macromolecular structure. Additionally, the selectivity of the solvent towards a particular monomer can be used to manipulate the morphology. In fact, the solvo-phobic segments of the chains are induced to minimize their contact with the solvent by forming micellar structures, while the solvo-philic components remain exposed. Thus, by properly designing the chains with a solvo-phobic endblock or midblock, even layering is achieved [26,84,86,92,95,96].

Although the ability of macromolecules to self-assemble has long been known, the dynamics that govern the final morphology are still under debate [26,84,87]. In this context, various theoretical approaches have been proposed over the years, but the complex multiscale relationship between composition and structure often requires molecular-level data that are typically not available. For this reason, simplified models have been developed [26,96,98,99,100,101,102,103,104,105,106,107,108].

On the other hand, in solvent-free processes, the microstructure is mostly determined by the balance between the orientation, relaxation and crystallization dynamics of the macromolecules through the selection of the right combination of materials, processing technique and parameters, and they can therefore be considered as top-down approaches [5,13,109]. Additionally, structuring is based on melt blending and consists of applying an external mechanical field to the material. For this reason, the microstructure can often be referred to as an external-field-determined morphology [2,5,13,14,20,26,89,90,91].

### 1.2. Solvent-Free Production of All-Polymer Materials

Considering all-polymer materials containing semi-crystalline polymers, the morphology in the solid state can be initially distinguished into amorphous and crystalline phases. However, the separation between the two is not sharp and can be influenced by several factors such as the chain topology and the presence of a flow field [5,110,111,112,113]. Specifically, the transformation of the molten state into the solid state under quiescent conditions results in the formation of crystals, and a three-dimensional spherulitic morphology of folded chains separated by the amorphous phase is obtained [5]. However, such a final microstructure can be properly modified by the application of a flow field, which interferes with the relaxation dynamics and thus affects the crystallization kinetics and the final crystallinity [2,5,13,40,109,114,115,116,117,118,119,120]. In particular, the main types of flow are elongational and shear flow, which are distinguished according to the mutual direction between the flow of the material and the velocity gradient inside the molten state [114]. As a consequence, different results are obtained by applying one or the other. The elongational flow has the ability to promote the orientation and even stretching of the macromolecules in the direction parallel to the flow of the material, thus promoting the formation of an anisotropic morphology. On the other hand, such an orienting ability is less effective for shear flow, and the isotropy of the microstructure can be largely maintained in this second case [114,117,121,122,123,124]. 

In addition, the final morphology is also influenced by the isothermal or non-isothermal condition in which the material is processed. In fact, the rate at which a material is cooled affects its rheological response, and, as a consequence, the resulting effect of the flow field may be different from that expected under isothermal conditions. Further, the formation of crystals may occur prematurely, taking into account both the contribution of the flow in promoting the crystallinity and the decreasing temperature [109,114,120,125].

All of this must be taken into account, especially in industrial-scale processing, where a non-isothermal step is often present. For instance, compounding and injection molding are widely used industrial techniques that are mainly characterized by the application of shear flow. On the other hand, the role of elongational flow is also appreciated when the melt enters the die section in compounding or in the mold gate in injection molding [114,126,127,128,129,130,131]. Additionally, external flow fields can also be introduced, as in the case of the application of the elongational flow in blow molding, film blowing, film casting and fiber spinning [5,17,91,114,120,132,133,134,135,136,137,138,139,140,141,142,143,144,145,146,147,148,149,150,151,152,153,154,155].

**Figure 1:**
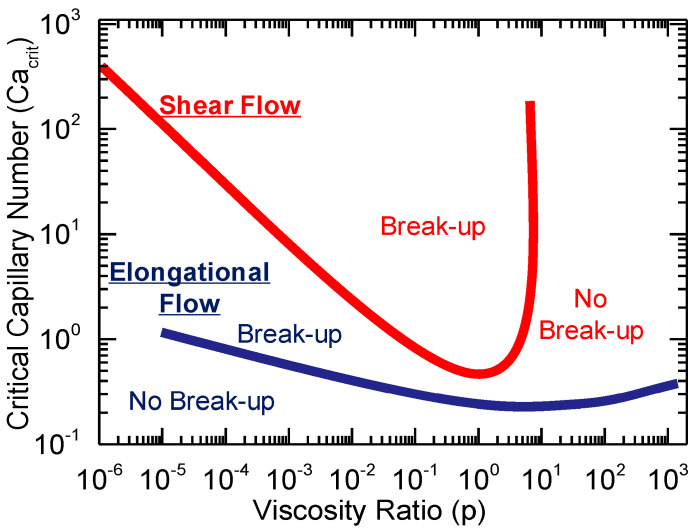
Critical capillary number as a function of viscosity ratio in polymer blends, based on Grace’s analysis [160]. Reprinted under CC BY 4.0 license.

On the other hand, when focusing on the melt processing of polymer blends, the role of the materials becomes apparent. In fact, in most cases they are thermodynamically immiscible in the molten state, so a heterogenous morphology characterized by droplet-like domains of the second phase dispersed in the matrix is commonly observed [156,157]. As a result, the applied shear or elongational flow can deform, orient or even induce the breakup of such droplets, depending also on the rheological properties of the polymers and the blend composition. Thus, the final shape and size of the secondary domains and hence the resulting microstructure can be tuned by knowing the intrinsic characteristics of the system [114,117,121,122,123,158,159]. Specifically, the effect of shear or elongational flow in determining the final morphology has been related to two dimensionless parameters that depend on the blend in question, namely, the viscosity ratio and the capillary number [114,160]. As shown in Figure 1, the breakup of the dispersed droplet is obtained when a critical capillary number is reached, and, considering a constant viscosity ratio, the value is determined by the type of flow applied. It was found that a lower capillary number is required in the presence of an elongational flow, because it is more effective in breaking the droplets. Also, in this case, it is possible to reduce the droplet size even for viscosity ratios higher than four, which, on the other hand, is a limitation that affects the shear flow [114,159,160,161].

**Figure 2:**
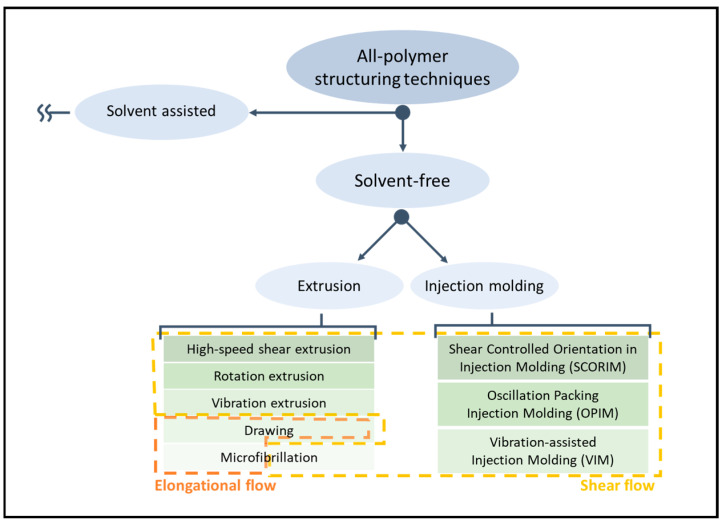
Schematic representation of the major solvent-free all-polymer structuring techniques and the corresponding active flow fields.

Considering the scenario described above, it seems clear that solvent-free structuring approaches are a valuable solution for tailoring the performances of all-polymer systems by exploiting melt-blending processing techniques and materials already used at industrial scale. Additionally, these techniques are less polluting of the environment and less hazardous for specialized workers compared to solvent-assisted ones due to the absence of a solvent. Nevertheless, the complexity of the relationship between processing parameters and microstructure has not yet been fully revealed due to the lack of knowledge on the evolution of macromolecular dynamics in the presence of an external field in real case scenarios and the lack of information on the actual thermo-mechanical field during melt blending, thus requiring further investigations in the coming years [109,162,163,164,165,166,167]. The scheme in Figure 2 shows a schematization of the primary manufacturing processes for producing polymer-based materials, distinguishing between those based on compounding and injection molding. Furthermore, the presence of shear or elongational flow is emphasized for each technique, helping to evaluate the current understanding of the relationship between processing parameters, microstructure and properties.

## 2. Extrusion

### 2.1. High-Speed Shear Extrusion

High-speed shear extrusion refers to a set of processing technologies (performed through the use of twin-screw extruders or, less commonly, internal mixers) in which, owing to high screw rotation speeds, a strong shear flow is applied to the molten material, typically inducing some microstructuring phenomena. The shear rate is usually between 200 and 750 s^−1^, but values up to 1650 s^−1^ have also been reported [168,169,170,171].

From an experimental perspective, the screw rotation speed is one of the most significant parameters in determining the shear rate. The value of the rotation speed usually exploited is about 1000 rpm [168,169,172,173] and can reach up to 4000 rpm [171,174,175]. However, structuring ability is observed even at lower screw speeds, starting from 400–500 rpm [168,169,170,173,176,177,178]. From a general point of view, such technology is quite simple in terms of equipment, considering that it requires an extruder (or an internal mixer) with a suitable engine and no additional components.

#### 2.1.1. The Role of the Screw Speed

It has been demonstrated that the microstructural modifications induced by high-speed shear extrusion are effective in increasing the mechanical properties of pure polymers. For instance, Zhang et al. [177] processed high-density polyethylene (HDPE) at 100, 500, 900 and 1200 rpm. From the characterization of the mechanical properties, it was appreciated that the increase in the screw speed from 100 to 1200 rpm resulted in the enhancement of the tensile strength from 32.0 to 46.7 MPa, respectively. Additionally, the corresponding elongation at break abruptly decreased from 562.8 to 29.9%. Thus, the application of a high shear to the HDPE introduces an overall self-reinforcing effect. This behavior is related to the macromolecular organization achieved due to the compounding at high screw speeds that results in the formation of oriented crystals. Additionally, the rheological characterization of the HDPE processed at the high screw speeds revealed a solid-like gel behavior, which was related to the presence of a three-dimensional network resulting from an increase in entanglement density. The authors [177] explained this unexpected phenomenon by taking into consideration different factors. Firstly, due to the very short residence time of the material inside the extruder, the macromolecules are exposed to the shear responsible for deformation for a short time, resulting in a reduced detangling effect. Furthermore, increasing the applied shear force enhances interactions between macromolecules, raising the probability of collision between chains and promoting entanglement formation. Also, high-shear processing has been shown to favor macromolecular diffusion, which contributes to entanglement formation. Considering all these factors and the final properties obtained, it was concluded that entanglement density increased during extrusion. Lastly, decreasing the molecular weight (MW) of the chain segments between two subsequent entanglements by increasing the screw speed was related also to denser packing, which resulted in the formation of packed oriented crystals. On a micrometric scale, this induces the formation of finer crystalline structures as compared to those obtained in HDPE processed at lower screw speeds. On the other hand, the decreased MW of the segments between the entanglements negatively affected the overall crystallinity content. Nonetheless, the tensile strength is positively affected by the increase in both the packing and orientation of the refined crystals. Thus, the anisotropy prevails with the lowering of the crystallinity in determining the final mechanical properties.

**Figure 3:**
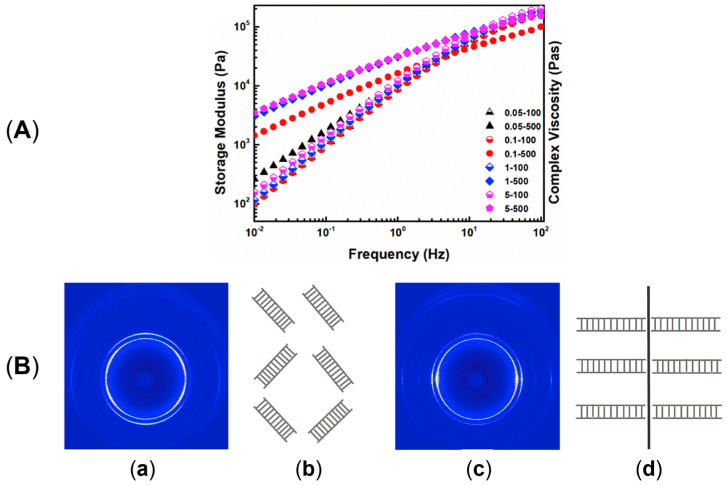
(**A**) Storage moduli of HDPE containing 0.05, 0.1, 1 or 5 wt% of UHMWPE and compounded at 100 or 500 rpm. (**B**) Two-dimensional WAXD patterns of HDPE\UHMWPE with 0.1 wt% of UHMWPE processed at (**a**) 100 rpm and (**c**) 500 rpm. The corresponding schematic representations of the crystalline structures are shown in (**b**) and (**d**), respectively [176]. Reproduced with permission from Elsevier Science Ltd., 2018.

Additionally, as assessed by Zhang et al. [176], such oriented crystals can be exploited for the formation of shish-kebab structures in PE homopolymer blends when ultra-high-molecular weight polyethylene (UHMWPE) is introduced. In the study, the performances of PE blends containing 0.05, 0.1, 1 and 5 wt% of UHMWPE were compared, considering 100 and 500 rpm as screw speeds. The evolution of the material microstructure was assessed through rheological analyses. As is observable from the storage moduli curves of the materials shown in Figure 3A, the blends compounded at 500 rpm and containing a minimum of 0.1 wt% of UHMWPE show a rheological response that is representative of the presence of a three-dimensional entangled network inside the materials. Additionally, the processing at high screw speeds induced the formation of shish kebabs. In fact, as can be appreciated in Figure 3B, these hierarchical crystalline structures (in which UHMWPE forms the shish, while HDPE constitutes the kebabs) are clearly distinguished in the samples processed at 500 rpm but not in those obtained at 100 rpm. Actually, in the latter case, the presence of crystals of HDPE distributed in a preferential direction is highlighted, indicating that, even if a structured anisotropic morphology was obtained at both screw speeds, the formation of the shish kebab only occurred at higher values. This feature was explained considering that the shear flow applied on the molten UHMWPE at 100 rpm was not enough to promote the alignment and orientation of its long chains in order to form the shish structure.

As will be detailed below, the structuring effect of high-speed shear extrusion on immiscible blends impacts the reduction in the droplet size and the achievement of a more homogeneous distribution in the matrix when compared to the same material processed at a lower screw speed. As a result, the ductility of the resulting materials is promoted owing to the smaller dispersed domains. In particular, the relation between the applied shear rate and the resulting droplet radius can be described with different mathematical models [169,179,180,181,182,183,184]. For instance, of particular importance is the one deriving from the work of Taylor [185,186] and based on the capillary number, which takes into consideration the effect of hydrodynamic stresses on the deformation of dispersed particles [183,187,188]. Specifically, the model is described with Equation (1) [17]:

(1) $$Ca=\frac{\eta_{m}\;\dot{\gamma }{\;R}_{Ca}}{\gamma_{12}}$$

where Ca is the capillary number, η_m_ is the viscosity of the matrix, $\dot{\gamma }$ is the shear rate, R_Ca_ is the droplet radius and γ_12_ is the interfacial tension between the two phases [17]. In addition, from Equation (1), Wu [189] developed an empirical relation to calculate the droplet radius as expressed by Equation (2):

(2) $$R_{Wu}=\frac{\gamma_{12}}{\dot{\gamma \;}\eta_{m}}\;4\;{\left(\frac{\eta_{d}}{\eta_{m}}\right)}^{\pm 0.84}$$

where R_Wu_ is the droplet radius calculated with the model of Wu, η_m_ and η_d_ are the viscosities of the matrix (m) and the dispersed phase (d), γ_12_ is the interfacial tension between the two phases, and $\dot{\gamma }$ is the shear rate [169]. 

Lastly, Serpe [7] developed a further model introducing the phase volume, which is related to the probability of collision between the particles [169] and is described by Equation (3):

(3) $$R_{Serpe}=\frac{\gamma_{12}}{\dot{\gamma \;}\eta_{m}}\frac{{\left(\frac{\eta_{d}}{\eta_{m}}\right)}^{\pm 0.84}}{\left(1-4{\left(\varphi_{d}\varphi_{m}\right)}^{0.8}\right)}$$

where R_Serpe_ is the droplet radius in accordance with Serpe’s model, γ_12_ is the interfacial tension between the matrix and the dispersed phase, $\dot{\gamma }$ is the shear rate, η_m_ and η_d_ are the viscosities, and φ_m_ and φ_d_ are the volume fractions of the matrix (m) and the dispersed phase (d) [169].

**Figure 4:**
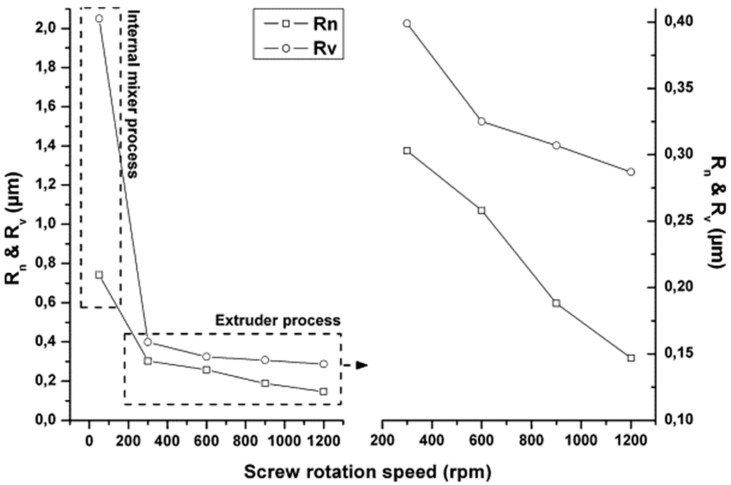
Number average (R_n_) and volume average (R_v_) radii of plasticized starch domains as a function of the screw speed in PA12-based blends [169]. Reproduced with permission from Elsevier Science Ltd., 2012.

The evolution of the number average radius (R_n_) and volume average radius (R_v_) of the droplet of 30 wt% plasticized starch in a 70 wt% Polyamide 12 (PA12) matrix compounded at 50, 300, 600, 900 and 1200 rpm was studied by Teyssandier et al. [169] and is shown in Figure 4. The corresponding shear rate for the lowest screw rotation speed is 40 s^−1^, while the values range from 200 to 750 s^−1^ when the screw speed increases from 300 to 1200 rpm. As can be clearly observed, a progressive reduction in Rn and Rv as a function of the rotation speed was documented. In particular, a R_n_ value of 0.742 μm was calculated for the material processed at 50 rpm, while for those obtained at 300 rpm the value was 0.300 μm. Additionally, the lowest R_n_ of 0.150 μm was achieved for 1200 rpm.

As a consequence, the elongation at break enhanced from 80% for the blend produced at 50 rpm (shear rate equal to 40 s^−1^) to 370% for those obtained at 1200 rpm (shear rate equal to 750 s^−1^). This behavior was related to the increase in the specific contact surface between the two polymeric phases due to the reduction in the dimensions of the dispersed phase domains. Also, this refinement of the blend morphology led to an increase in the impact strength from about 4 kJ/m^2^ in the material compounded at 300 rpm to 7 kJ/m^2^ when the exploited screw speed corresponded to 1200 rpm [169].

**Figure 5:**
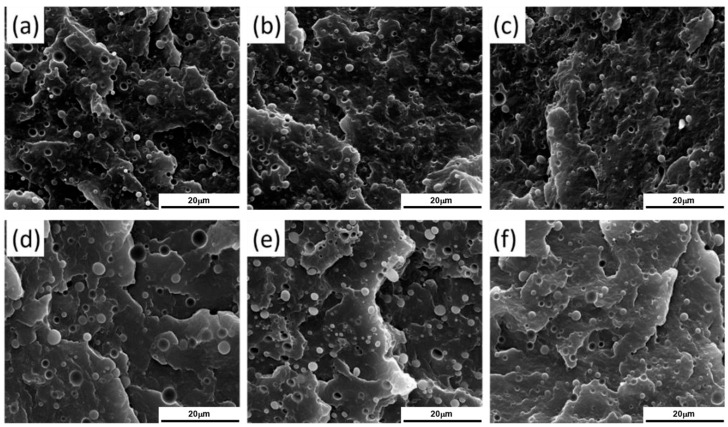
SEM micrograph of 90 wt% PP\10 wt% PLA blends. The materials were produced with different temperatures and screw speeds: (**a**) 190 °C—100 rpm; (**b**) 190 °C—500 rpm; (**c**) 190 °C—900 rpm; (**d**) 220 °C—100 rpm; (**e**) 220 °C—500 rpm; (**f**) 220 °C—900 rpm [170]. Reproduced with permission from Elsevier Science Ltd., 2018.

A similar behavior was observed for 90 wt% Polypropylene (PP)\10 wt% Poly(lactic acid) (PLA) blends processed at different screw speeds and at two temperatures (namely, 190 and 210 °C) [170]. In fact, in Figure 5 it can be noticed that, either at 190 (inserts a–c) or 220 °C (inserts d–f), a decrease in the droplets’ diameter is achieved by increasing the screw speed from 100 (inserts a and d) to 500 (inserts b and e) or 900 rpm (inserts c and f). Accordingly, the applied shear rate rose from approximately 100 to 600 s^−1^. Specifically, focusing on the materials obtained at 100 or 900 rpm, the average size of the PLA domains decreased from 1.09 μm to 0.68 μm at 190 °C and from 2.16 μm to 1.53 μm at 220 °C. The evolution of the blend morphology was attributed to the high shear that promotes the breakup of the PLA domains and, at the same time, hinders the coalescence phenomena due to the reduction in the residence time and thus in the period of contact between the molten domains of the dispersed phase.

However, the impact of the morphological changes on the resulting mechanical properties was limited. In fact, considering the elongation at break, an enhancement from 175 to 188% was observed for the blends compounded at 190 °C and from 121 to 156% for those produced at 220 °C, when the screw speed was 100 or 900 rpm, respectively, as the shear rate increased from about 100 to 600 s^−1^. Also, no significant variation in yield strength and impact strength was observed.

Differently, the importance of the droplet size reduction in increasing the ductility was highlighted by Shimizu et al. for different blends where the dimensions of the dispersed droplets were on the nanometer scale [171,172].

In particular, in the case of blends containing 80 wt% of polycarbonate (PC) and 20 wt% of polymethyl methacrylate (PMMA), a decrease in the domains’ dimensions from 2 μm–200 nm to less than 50 nm was achieved by passing from 300 to 2250 rpm, with a remarkable modification of the material morphology [171]. This last, in turn, promoted an impressive increase in the ductility from 22 to 118% for blends obtained at 300 or 2250 rpm, corresponding to a shear rate of 220 and 1650 s^−1^, respectively. The phenomenon was likely due to the enhancement of the compatibility between the polymers induced by the increase in the specific contact surface resulting from the reduction in the droplet size [168,170,171].

In the studies discussed so far, the beneficial effect of the screw speed on decreasing the droplet size was highlighted. However, some studies [168,173,178] evidenced deviations from the models, and this was related to the degradation of the matrix due to the exceedance of the optimal screw speed [168,173] or to the polymers’ reciprocal content [178].

**Figure 6:**
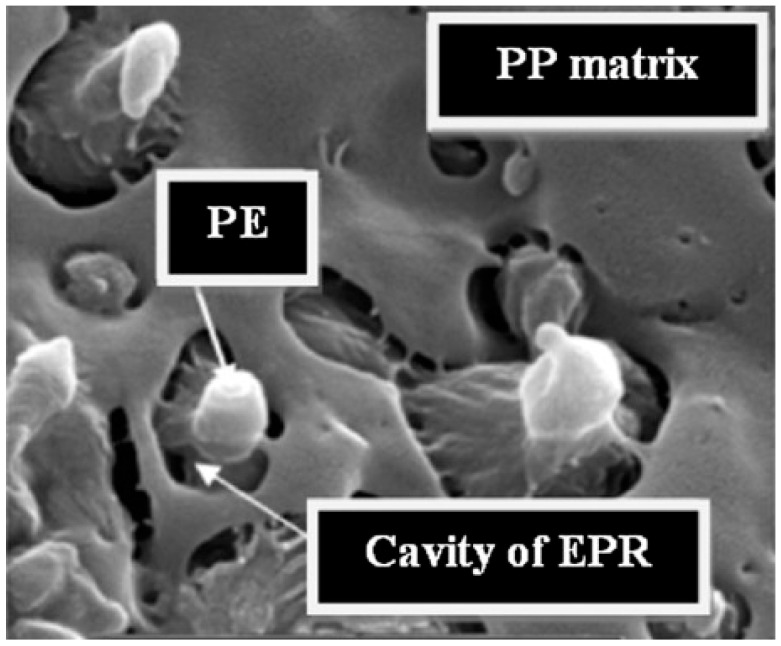
SEM micrography of core–shell layered PE\EPR droplets observed in the PP\EPR\PE ternary blend compounded at 600 rpm [168]. Reprinted with permission from John Wiley and Sons, 2014.

For instance, Louizi et al. [168] analyzed the morphology in a 62 wt% PP\20 wt% PE rubber\18 wt% PE (PP\EPR\PE) ternary blend compounded at 300, 600, 800 or 1200 rpm, corresponding to maximum shear rates of 220, 400, 500 and 750 s^−1^, respectively. In all the cases, the dispersion of the PE\EPR domains in the PP matrix was appreciated. It was shown that at 300 rpm the blend morphology was characterized by coarse irregular droplets with a marked tendency to coalescence, while at 600 rpm a decrease in the domains’ size was achieved, along with a modification of the shape of the dispersed domains. However, the most interesting observation is shown in Figure 6. In fact, core–shell layered PE\EPR droplets were identified in the blend compounded at 600 rpm. Furthermore, increasing the screw speed resulted in an increment in the radius of the dispersed domains, which increased from 0.2 µm for blends processed at 600 rpm to 1 µm for those compounded at 1200 rpm. The authors suggested that this phenomenon may be due to the β-scissoring of PP and to the crosslinking between PE and EPR. Both are related to thermo-mechanical degradation, which may affect the polymers during compounding at such high shear rates. In addition, the coalescence of the droplets due to the presence of chain interdiffusion at the droplet interfaces has been suggested to be responsible for the large polydispersity of the dispersed domains size in the blends processed at the maximum shear rates of 500 and 750 s^−1^ [168].

The ductile behavior of the blends evolved accordingly. In particular, the elongation at break for the materials processed at 300 rpm (or 220 s^−1^) was 200%, and the value increased up to 480% for the material obtained at 600 rpm (corresponding to a shear of 400 s^−1^). Such improvement was related to both the refinement of the droplet size and the core–shell structures formed in these conditions. Furthermore, the elongation at break decreased to 310 and 170% for the blends produced at 800 and 1200 rpm, respectively [168]. A similar trend was observed for the impact strength. Specifically, the value of 40 MPa increased up to 70 MPa when the screw speed of 300 or 600 rpm was considered. Then, the impact strength abruptly decreased to 35 and 30 MPa for the blends processed at 800 and 1200 rpm, that is, for shear rates of 500 and 750 s^−1^, respectively [168]. The latter behavior was related to the effect of the droplet dimensions, since larger particles promote crack coalescence, thus negatively affecting the impact properties [178].

Also, Raj et al. [173] documented that the degradation of the matrix during processing is responsible for the coarsening phenomena observed in PLA\PA12 blends containing 30 wt% of polyamide and processed at 200, 500, 800 or 1100 rpm. In fact, the average diameter of the domains decreased from 0.72 μm at 200 rpm to the minimum of 600 nm at 800 rpm. Then, the size increased again to 1.1 μm in the materials compounded at 1100 rpm. As far as the mechanical properties of the blends are concerned, the elongation at break enhanced from 154.5 to 224.2% in the materials processed at 200 and 800 rpm, respectively. Additionally, the value decreased to 18.1% for the system produced at 1100 rpm. Similarly, the improvement in the impact strength from 33.5 to 48.2 kJ/m^2^ reflected the droplet size reduction, while the lowering to 31.8 kJ/m^2^ in the blend produced at 1100 rpm highlighted once again the degradation of the matrix [173].

#### 2.1.2. The Role of the Matrix-to-Second-Phase Content

Yu et al. [178] associated the maximum achievable size reduction with the composition of the blend. In their study on PE\PA12 blends compounded at 100 or 500 rpm, the effect of three different reciprocal contents on the material morphology was assessed. In particular, a decrease in the average droplet diameter with an increase in the screw speed was observed only in the materials containing 5 or 10 wt% of PA12, for which the value lowered from 1.05 to 0.53 μm and from 1.11 to 0.60 μm, respectively. Conversely, no size reduction was appreciated for the blend containing 20 wt% of PA12. The different behavior was related to the content of the polyamide, which, in the latter case, was high enough to favor the coalescence (due to the increased probability of collision between PA12 domains) over the size reduction driven by the applied shear [178]. Additionally, it is interesting to note that the composition limit seems to be related to the polymers involved in the blend. In fact, no evidences of issues regarding size reduction were reported by Raj et al. [173] for the system of PLA\PA12 containing 30 wt% of polyamide.

Lastly, the elongation at break was affected by the polyamide content in PE\PA12 blends [178]. In particular, it increased from 90.5% to 306.9% when the material containing 5 wt% of PA12 was compounded at 100 or 500 rpm. Additionally, an increase in strain at break as a function of screw speed was observed in the presence of 10 wt% polyamide, with values rising from 70.3% at a low rotation speed to 224.2% at a high rotation speed. On the other hand, the material containing 20 wt% of PA12 was not affected by the decrease in the average diameter, and, as a consequence, lowering of the elongation at break was observed instead. Specifically, the value decreased from 64.6 to 54.7% when the parameter was set at 500 rpm rather than 100 rpm. Similarly, an improved impact strength was appreciated in the blends containing 5 or 10 wt% of PA12 with the increase in the screw speed: it enhanced from 14.5 kJ/m^2^ to 22 kJ/m^2^ and from 9.3 kJ/m^2^ to 12.7 kJ/m^2^, respectively, while for 20 wt% no variation was observed.

### 2.2. Rotation Extrusion

**Figure 7:**
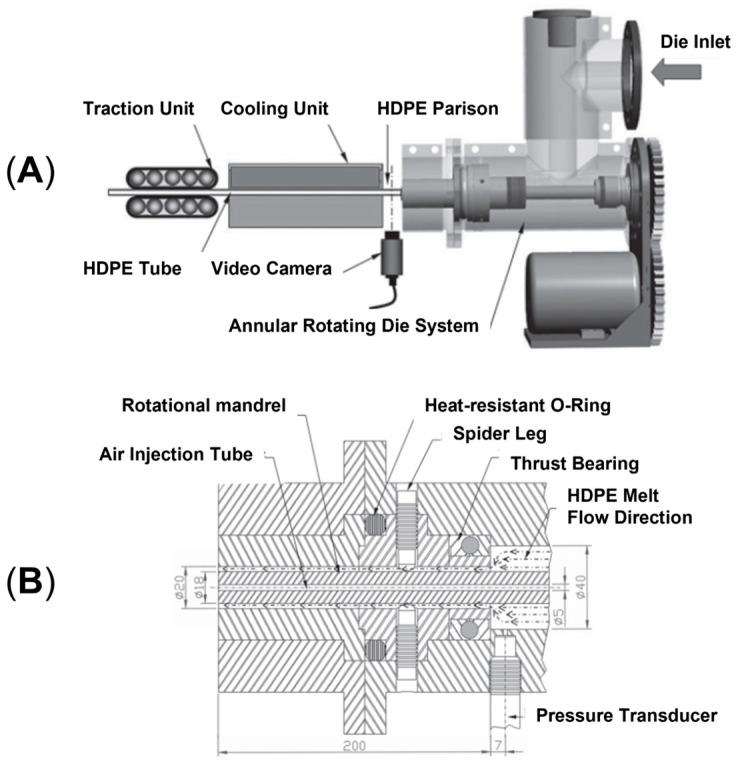
Schematic representation of the (**A**) instrumental setup used for rotation extrusion and (**B**) a cross-section of the annular rotating-die head [196]. Adapted with permission from Springer Nature 2014, SNCSC.

In rotation extrusion, the structuring effect on the melt polymer is obtained owing to the shear flow resulting from the superposition of two flow fields. In particular, a first shear flow is applied on the material in the direction parallel to the extrusion, while, in addition, a second shear field oriented perpendicularly to the extrusion direction is imposed on the molten material in correspondence to the die, due to the presence of annular rotating equipment with an external steady case and an inner rotating mandrel. A schematic representation of the instrumental setup and the detail of the cross-section of the annular rotating die are given in Figure 7A,B, respectively. A helical flow results from the superposition of the two flow fields. It has been shown that the resulting flow is able to promote macromolecular orientation phenomena, allowing microstructuring [45,190,191,192]. Additionally, the potentiality of the technique appears clear considering polymers like PE and PP, in which the formation of shish kebabs can be reached. In fact, by exploiting rotation extrusion for polyolefin processing, such hierarchical structures can be oriented away from the extrusion direction, ultimately resulting in the modification of the mechanical performance [193,194,195,196,197].

For this reason, this technology is of particular interest for the production of pipes. In the conventional approach, the extruded material reaches the final shape owing to an annular steady die and an inner air flux that inflates the tube. As a result, the macromolecules are preferentially aligned in the axial direction and the axial tensile strength of the pipe is higher than the circumferential one, i.e., the so-called hoop tensile strength. However, this is not desired in pipe applications because the radial internal stress faced by these components is twice the axial one; therefore, higher performance in the radial direction would be preferred. For this reason, great effort has been spent in the last few years to increase the hoop strength that, in the end, determines the operating pressure limit. In this regard, rotation extrusion represents a valuable processing technology [45,51,190,191,192,193,194,195,196,198]. On top of that, the structured macromolecular organization obtained with this technique also solves a further problem negatively affecting pipes’ operating conditions, namely, the heat resistance. In fact, high-temperature fluids are often present in pipe applications and may promote the evolution of the microstructure toward a more isotropic organization that negatively affects the hoop strength over time [195].

#### 2.2.1. The Role of Screw and Mandrel Rotation Speeds

**Figure 8:**
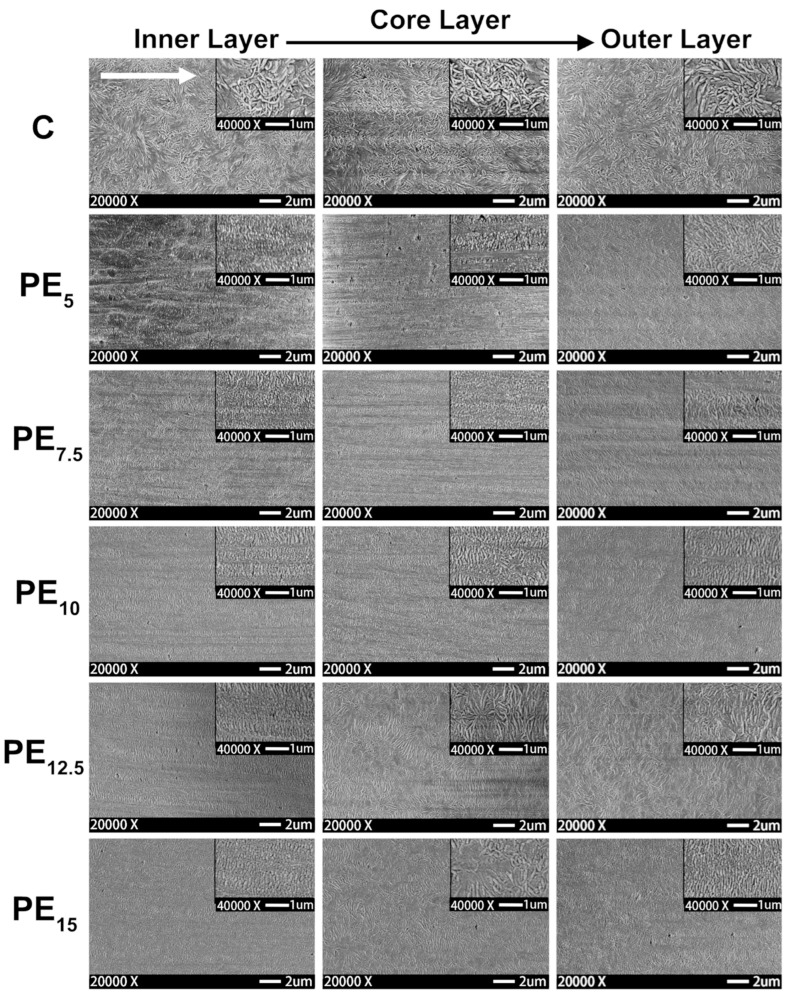
SEM micrographs of the section of the pipe of HDPE processed with mandrel rotation at 0, 5, 7.5, 10, 12.5 or 15 rpm. The white arrow indicates the flow direction [195]. Reproduced with permission from Elsevier Science Ltd., 2019.

Studies of the effect of rotation speed on microstructure are often performed with a particular piece of equipment named a Rotational Shear System (RSS) [193,195]. It is a batch system, in which a limited amount of material is inserted in a pipe-shaped rotating mandrel device. As soon as the material is placed in the mold, the mandrel starts to rotate at a constant speed while a cooling ramp lowers the temperature of the die. An RSS allows better control of the processing conditions without affecting the reliability in simulating the actual rotation extrusion process. For instance, this approach was exploited to assess the effect of different screw rotation speeds on the microstructures and final properties of a commercial bimodal molecular weight HDPE [195]. The mandrel rotation was set at 5, 7.5, 10, 12.5 and 15 rpm. The morphology obtained in the thickness of the pipe’s wall is shown in Figure 8. In particular, the inner layer refers to the material in contact with the rotating mandrel, while the outer layer is that next to the external mold. The core layer refers to the polymer in between.

It is important to note that, even though a single cooling system regulates the temperature of both the mandrel and the wall of the rotating component, a displacement in the cooling rate of the two was evidenced in more than one study [191,195]. In particular, the reduction in the temperature of the outer walls resulted in a lower cooling rate for the mandrel surface, and this affected the evolution of the microstructure in certain processing conditions.

Scanning Electron Microscopy (SEM) micrographs of a pipe produced without rotation (Figure 8, first row) highlight an isotropic dispersion of spherulites. In this case, no shear was applied; thus, the final microstructure is characterized by random lamellar growth. In addition, the effect of the mandrel cooling can be appreciated. Specifically, the size of the spherulites is higher in the core layer and decreases in the inner and outer ones, due to the higher colling rate of the external material in contact with the metallic die.

When mandrel rotation occurred, the evolution of the microstructure toward the formation of anisotropic shish kebabs was observed. In particular, their presence depends on the mandrel rotation speed, which also affects their orientation. Specifically, the shishes are more parallel to the axial direction, while the kebabs are perpendicular. However, an increase in the displacement toward the radial orientation was observed with the enhancement of the rotation speed due to the superposition of the axial and hoop drag flows. In fact, a homogeneous distribution of shish kebabs in all the pipe’s thickness was observed only at 7.5 rpm (Figure 8, third row). Conversely, at 5 rpm (Figure 8, second row) these hierarchical structures were distinguished only in the inner and core layers, while the outer one only contained ordered-lamellae structures. Otherwise, a further increase in the mandrel rotation speed up to 12.5 and 15 rpm led to a progressive evolution of the anisotropic shish kebab in ordered lamellae and, lastly, in spherulites.

The evolution of the microstructure was explained considering two factors, namely, the reaching of the critical strain rate in the melt and the shear heating. Considering the “coil-stretch transition” theory, a threshold strain rate is required in order to obtain the minimum stretching and orientation level of the macromolecules for the formation of shish-kebab structures. In an RSS, the motion is imposed on the material starting from the inner layer. Thus, the shear rate applied to the material decreases as the radial distance from the axes increases. This means that at 5 rpm the mandrel speed is not high enough to ensure the crossing of the strain rate threshold throughout the pipe’s thickness, specifically in the external layer, where the isotropic morphology is observed. The critical value is exceeded throughout the material at 7.5 rpm, where the peculiar hierarchical microstructure is appreciated throughout the thickness.

The second phenomenon, named shear heating, explains the progressive evolution of the microstructure toward a less ordered configuration with the increase in the mandrel rotation speed. This involves the progressive rise in the material’s temperature due to the increased shear, which promotes the relaxation of the macromolecules. For this reason, the loosening of orientation in favor of a more disordered conformation is observed. However, it is interesting to note that in the sample obtained at 12.5 rpm, shish kebabs could still be appreciated in the inner layer. This is due to the faster cooling rate characterizing this section, as mentioned above.

The obtained morphologies strongly determine the final properties. In particular, the hoop tensile strength was related to the presence of the oriented shish kebabs. In fact, compared to the pipes extruded with the mandrel at rest, the tensile strength of those obtained at 7.5 rpm increased to 338%. The further enhancement of the rotation speed resulted in a decrease in the mechanical properties, due to the already discussed evolution of the material microstructure, involving the formation of spherulites in the core and outer layers.

The increase in the tensile strength at 7.5 rpm is explained by the dynamics occurring inside the polymer when the shish-kebab microstructure is present. In fact, these oriented structures are interlocked, and this hinders the macromolecules’ sliding, the quick stretching of the coiled chains and, ultimately, lamellae slippage. In addition, the shish structures are made of extended chains; thus, the ductile deformation associated with the disentanglement and chain slippage is largely suppressed and, in fact, a brittle fracture behavior is observed. For the above reasons, the increase in the tensile strength is associated with the decrease in the elongation at break, which is lowered from about 170% to 20–30% when the mandrel is in motion.

In addition, the thermal resistance was also improved. Specifically, the Vicat Softening Temperature (VST) increased from 76.7 to 103.5 °C for the mandrel rotation speed of 7.5 rpm. In general, a VST higher than 100 °C was obtained for all the samples in which the shish-kebab structures were present. As already mentioned, the presence of the aligned shishes and the interlocked kebabs forms a dense fiber-like network that enhances not only the mechanical properties but also the thermal resistance of the overall material.

The effect of the mandrel rotation speed on the final properties was also evaluated by Nie et al. [191] with Slow Crack Growth (SCG) analysis or cone tests. In this study, PE pipes were obtained with mandrel rotation speeds of 0, 5, 10 and 20 rpm. The sample produced at 0 rpm was characterized by the lowest crack initiation time of 27 h and the highest crack growth speed. The two parameters improved when the speeds of 5 and 10 rpm were exploited. However, with a further enhancement to 20 rpm, the crack propagation resistance decreased and was comparable to that obtained at 5 rpm. The improvement in the craze resistance was related to the enhancement in the orientation of the macromolecules in the hoop direction owing to the superposition of the two flows. In fact, the multi-axial orientation of the molecular chains was reached. This resulted in a decrease in the actual stress acting on each chain; thus, the disentanglement speed lowered and the SCG increased [197,199]. On the other hand, the decrease in the SCG resistance at 20 rpm was also in this case related to the shear heating. In fact, the transition of the oriented microstructure back to random coils promoted by the relaxation of the chains reduced the crazing resistance.

Similarly, the lowering of the tensile strength over a certain mandrel rotation speed was observed also in the continuous pipe production process [198]. In particular, the hoop tensile strengths of the pipes obtained with rotation speeds of 5, 10, 15 and 20 rpm were compared to those of the samples extruded with no motion. The tensile tests highlighted the maximum increase in the hoop strength up to 27.9 MPa when the rotation speed was 10 rpm, considering the value of 17 MPa for the pipe produced with no rotation involved. Additionally, in accordance with the aforementioned studies, a lowering to 22 MPa was measured in the pipes produced at 15 or 20 rpm. Such worsening of the hoop strength was attributed to crystalline defects resulting from the application of excessive stress on the material during crystallization.

#### 2.2.2. The Role of the Annealing Treatment

**Figure 9:**
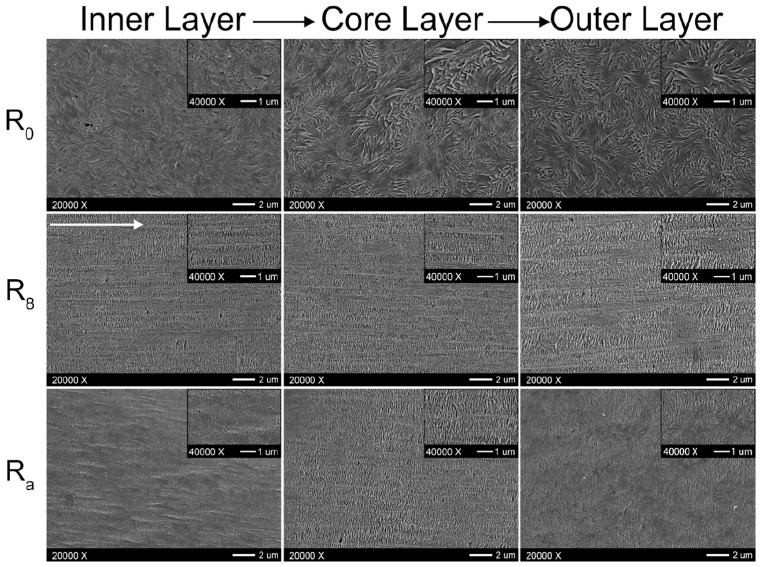
SEM micrographs of HDPE pipes throughout the wall thickness. First column: layer in contact with the mandrel; second column: central layer; third column: layer in contact with the die. First row: mandrel at 0 rpm; second row: mandrel at 8 rpm; third row: sample obtained with the mandrel at 8 rpm and subsequent annealing process at 125 °C for 40 min [193]. Reproduced with permission from Elsevier Science Ltd., 2020.

Furthermore, the effect of an annealing treatment on the microstructure of a commercial bimodal MW HDPE was studied with an RSS, and the improvement in the thermal properties along with the mechanical characteristics was assessed [193]. In Figure 9 the SEM micrographs of the samples produced without motion, with 8 rpm as the mandrel rotation speed and after annealing at 125 °C for 40 min are reported. In accordance with the already described studies, the material extruded without mandrel rotation shows an isotropic spherulitic microstructure, while the processing with 8 rpm of rotation induced a shish-kebab anisotropic morphology that characterizes all three layers. As a result of the annealing step, the inner and outer layers show a decrease in the shish-kebab content, while a clear shish-kebab frame can be observed in the core layer. The evolution of the microstructure was explained considering both the proximity to the melting point and the thermal stability of the oriented structures. In fact, at such a high temperature, the relaxation of the macromolecules is promoted and the thinner lamellae tend to melt more easily. However, compared with the core layer, where coarser shishes and kebabs are present, the oriented structures are thermally more stable; thus, the melted polymer tends to orientate according to the crystalline domains retained. As a result, the increase in the thickness of the kebab lamellae, the enhancement of their lateral size and the improvement in the interlocking degree are obtained.

The most important effect of the evolution of the microstructure due to the annealing treatment concerns the improvement in the thermal properties in terms of VST and thermal conductivity. In both cases, the enhancement is explained considering the improved intimacy reached between the polymer chains. Considering VST, it increased by 10.7 °C (up to 112.4 °C) after the annealing, and this phenomenon was related to the formation of a network-like structure of oriented shish kebabs owing to the increase in the interlocking degree of the kebabs. This morphology also promoted the transmission of thermal energy, leading to an increase in the thermal conductivity (either in-plane or out-of-plane) as compared to the material characterized by an isotropic crystalline structure. On the other hand, the annealing treatment did not significantly affect the mechanical properties in terms of both hoop tensile strength and axial strength.

Lastly, the effect of the presence of 1 wt% of UHMWPE on the microstructure of PE pipes produced via rotation extrusion at a constant mandrel speed was deepened [200]. More specifically, it has been shown that the presence of UHMWPE promoted the formation of the shish-kebab structures when compared to the neat PE. In addition to the achievement of denser kebabs, the orientation away from the axial direction also improved.

The more packed hierarchical structure in the radial direction resulted in an increase in the hoop strength owing to the hindering in the crack propagation. In fact, SCG measurements highlighted that in the presence of UHMWPE better performances were obtained. In particular, the crack initiation time increased from 43 to 92 h, while the crack growth rate decreased from 0.16 mm/h to 0.11 mm/h. This is in accordance with the previous observations [193], in which more packed kebabs were related to the enhancement of the tensile performances.

#### 2.2.3. The Role of the Die Temperature

The importance of the macromolecular relaxation dynamics in the tailoring of the final microstructure of rotation-extruded polyethylene pipes also emerged from the analysis of Nie et al. [201] on the effect of the variation in the temperature of the die on the resulting mechanical performances. In particular, for the processing performed at 170 and 190 °C, a hoop strength of 31.8 and 31.4 MPa was measured, respectively, while the value decreased to 26 MPa for a die temperature of 150 °C and to 24 MPa at 210 °C. Such variation in the tensile behavior can be explained by the evolution of the microstructure at the different temperatures. In fact, at 210 °C the value was high enough to promote the relaxation of the chains toward the formation of an isotropic spherulitic morphology. On the other hand, despite the formation and orientation of shish kebabs being achieved at 150 °C, the temperature was close to the melting point and the application of the shear resulted in the formation of defected crystals. As a consequence, the final hoop strength was lower than that showed by the materials processed with a die temperature of 170 or 190 °C.

#### 2.2.4. The Role of the Nucleating Agent

Besides PE, PP is widely exploited in pipe production [45,51,190,194,202]. Its applications range from drinking water, transportation engineering and underground drainage. However, this material is affected by microstructural modification during conventional pipe extrusion that negatively affects its impact toughness [194,203]. Specifically, the formation of an isotropic α-spherulitic phase can be observed. To prevent its formation, isotactic polypropylene (iPP) along with β-nucleating agents are often used in conventional pipe production processes. In fact, the β phase is metastable and particularly effective in absorbing the impact energy due to the transformation in α crystals. This results in the formation of crazes inside the matrix and, consequently, in the enhancement of the toughness and impact resistance.

However, the drawback of this approach is the resulting decrease in the hoop strength of the pipe, which, being the most problematic point for this application, has to be mitigated. In this context, rotation extrusion was identified as a viable solution. In fact, also for PP, this processing technology allows promotion of the formation of shish-kebab structures away from the axial direction that, ultimately, cause an increase in the hoop strength. Additionally, considering that the morphology of the crystalline phase is influenced by the shape of the crystalline nuclei at the beginning of crystallization [15,203], the introduction of fibrous β-nucleating agents can also be exploited to further induce the formation of shish kebabs.

The importance of the simultaneous presence of mandrel rotation and a nucleating agent was clearly evidenced in the work of Nie et al. [45]. In particular, it was shown that the simultaneous application of mandrel rotation and the presence of a nucleating agent are mandatory for achieving stable shish-kebab crystalline structures well-oriented along the hoop direction. In fact, if the processing is carried out without mandrel rotation or in the absence of a nucleating agent, an isotropic microstructure involving α-spherulitic crystals is obtained. Interestingly, it has been demonstrated that the β-metastable phase is also achieved by applying mandrel rotation without a nucleating agent, testifying the formation of shish kebabs during the processing. However, without nucleating agents, these anisotropic crystalline structures tend to relax back in random coil conformation due to the high temperature, and the resulting microstructure is isotropic.

These differences in the microstructure caused significant variations in the materials’ mechanical behavior. Specifically, the application of the mandrel rotation in the samples containing the nucleating agent induced an increase in the impact strength from 9 to about 14 kJ, due to the high quantity of a β-crystalline phase. Furthermore, the hoop strength increased up to 15 kJ in the presence of well-oriented stable shish kebabs. Additionally, it was also shown that the hoop strength was significantly affected by the mandrel rotation speed, with a trend very similar to that already discussed for PE [51]. In particular, by comparing samples of iPP pipes produced through conventional extrusion or with increasing mandrel rotation speeds of 4, 8 and 12 rpm, the hoop strength enhanced from 21.3 MPa at 0 rpm to 30.0 and 34.0 MPa at 4 and 8 rpm, respectively. However, a further enhancement to 12 rpm resulted in a decrease in the tensile strength down to 26.7 MPa. This behavior was related to the unsteady flow of the polymer at such a speed, which induces the formation of microstructural defects, unfavorably affecting the mechanical performances.

The effect of a β-nucleating agent and, especially, its morphology on the development of the microstructure of iPP was also studied by Pi et al. [190], using 0.3 wt% of a dot-like or an irregular block-like nucleating agent. The obtained results demonstrated that an isomorphic β-spherulitic distribution was achieved in the presence of the dot-like agent, regardless of the application of mandrel rotation; otherwise, the formation of a highly oriented microstructure was observed for the materials containing the block-like nucleating agent.

The further characterization through SEM confirmed that this ordered microstructure was made of hybrid shish kebabs, where the nucleating agent formed the shishes and the β crystals formed the epitaxial kebabs, in accordance with what has already been described above [45].

**Figure 10:**
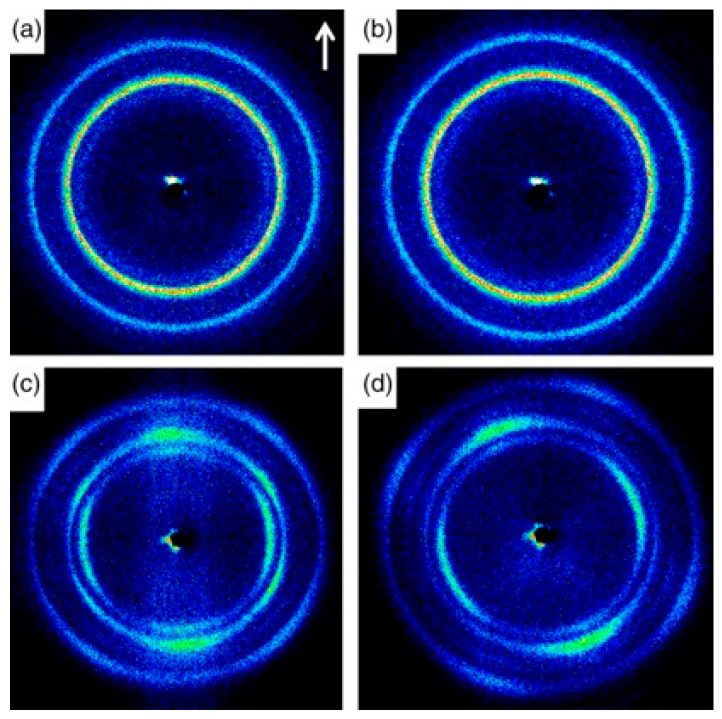
Two-dimensional WAXD of iPP obtained with (**a**) dot-like NA and no mandrel rotation, (**b**) dot-like NA and mandrel rotation, (**c**) block-like NA and no mandrel rotation, and (**d**) block-like NA and mandrel rotation. The white arrow indicates the axial direction [190]. Reprinted with permission from John Wiley and Sons, 2019.

Further, from the 2D WAXD patterns shown in Figure 10, the effect of the mandrel rotation on the orientation of the final microstructure can be appreciated. Specifically, contrary to what was observed by Nie et al. [45], no effect on the orientation was noticed when only spherulites were present (Figure 10b). On the other hand, tilting was clearly detected from the comparison with Figure 10c,d. Specifically, the orientation angle of the hierarchical microstructure obtained with mandrel rotation was quantified with the Herman equation as being 25°.

Also in this case, the presence of a β phase and anisotropy in the microstructure affected the mechanical behavior. In particular, according to [51], the impact strength was related to the quantity of the metastable phase and not to the mandrel motion. Specifically, the dot-like nucleating agent induced the formation of a higher quantity of β crystals (89.3 and 92.8% for the conventional and rotation processes) compared to the block-like one (86.4 and 84.4%, respectively), hence resulting in enhanced impact strength. Additionally, the anisotropic microstructure oriented away from the axial direction were confirmed to provide the best contribution in the hoop strength. That is, in the presence of the isotropic spherulitic distribution, 24 MPa and 23 MPa were reached with the steady or rotating mandrel, while the hoop strength enhanced up to 34 MPa when the shish kebabs were driven by the helical flow away from the axial direction.

The orientation of the shish kebabs away from the axial direction was achieved not only in iPP but also in polypropylene copolymers containing about 3.8 wt% of ethylene [202]. In particular, with the introduction of a nucleating agent and iPP as a crystallization promoter, the concentration of the mesomorphic phase increased from 0% to 51%. Additionally, by comparing the mechanical performances of the pipes extruded with the mandrel in motion at 6 rpm, the enhancement of the impact strength from 4 to 7 KJ/m^2^ was measured, along with an increase in the hoop strength from 23.7 to 28.9 MPa. This was due to the simultaneous presence of iPP and the NA, since both the properties decreased if only one of them was present. For this reason, the improvement in the mechanical features was attributed to the synergistic mechanism of the two in the formation of the shish-kebab structures. Specifically, the nucleating agent provides the fibrous nuclei forming the shishes, on which the β-phase kebabs of iPP macromolecules will grow. Further, such structures provide the brackets from which the β crystals formed by the copolymers will develop, eventually resulting in a highly anisotropic hybrid shish-kebab microstructure. Lastly, these hierarchical structures will orientate away from the axial direction thanks to the superposition of the axial and hoop drag flow owing to mandrel rotation.

Accordingly, the retention of the isotropic spherulitic microstructure in PP copolymers containing ethylene, with no addition of nucleating agents or iPP, was confirmed by Han et al. [204]. The study on the effect of the mandrel rotation speed and cooling rates on the morphology and mechanical properties of the neat copolymer confirmed that in none of the samples considered was the formation of an oriented microstructure observed, despite the variation in the processing conditions. However, the orientation of the macromolecules in the amorphous phase away from the axial direction was observed with the rotating mandrel. Nonetheless, a slight increase in the mechanical performances, attributed to melt structuring of the amorphous phase, emerged.

The orientation effect on the amorphous phase due to the mandrel rotation was evidenced also in polybutadiene-1 (PB) pipes, along with an increase in crystallinity and a decrease in the crystals’ defects [192]. In particular, in PB, the presence of shish kebabs was not observed for any mandrel rotation speed (ranging from 2 to 12 rpm), and this feature was attributed to the greater steric barrier toward the orientation of the macromolecules when compared to PE and PP, as well as to the long crystallization time of PB. Moreover, a further drawback is the higher time provided for the chains to relax in such a system. In fact, even if a slight lamellar orientation was present at the highest rate, the microstructure was predominantly spherulitic and isotropic. In addition, the orientation of the macromolecules is more easily retained in the amorphous regions [205], and this phenomenon was invoked for the observed improvement in the hoop tensile strength with the increase in the rotation speed. In particular, the value increased from 20 to 25 MPa with the increase in the mandrel rotation speed from 0 to 8 rpm. Further, also in PB, a too high mandrel rotation speed was associated with a decrease in the mechanical properties due to the formation of defects [51,192,198].

### 2.3. Vibration Extrusion

This approach is specifically meant to affect the density of entanglements owing to the extra shear applied on the material through the presence of an external vibrational field [14,206,207,208,209]. As a consequence, the increased energy transferred to the macromolecules promotes the conformational freedom that, ultimately, leads to a lower entanglement density [206,207,210]. As a result, vibration extrusion is effectively exploited for the compounding of polymers which, due to the high density of entanglements, are challenging to process with conventional solvent-free approaches. Furthermore, it was shown that the elastic turbulence and melt fracture was significantly lowered, while the overall throughput was increased by 50 to 100% [210,211,212,213,214,215,216,217,218]. Ultimately, the final morphology and surface quality, along with the resulting mechanical properties, are affected [206,207,208,210,211,214,216,217,219,220,221,222].

All the above is often referred as the physical effect of vibration [210,215,223,224]. Nevertheless, the application of an ultrasonic field can also result in a chemical effect, which has been recognized to induce a decrease in the polymer molecular weight and a consequent modification of the molecular weight distribution, due to degradation phenomena promoted during the processing [14,207,210,211,215,216,223,224,225]. However, in some cases the formation of long-chain radicals resulting from chain-scission reactions was profitably exploited for in situ compatibilization of immiscible blends [210,225,226].

Depending on the applied frequency, this processing technology is usually distinguished in “ultrasonic vibration extrusion” (hereafter referred to as ultrasonic extrusion) and “mechanical or oscillation extrusion” (named oscillation extrusion below) [14,206,211]. In the first case, the frequency is above 20 kHz, while for the former it is between 0 and 100 Hz [14,206]. The vibration field can be applied in both the longitudinal and the transversal directions. However, the former option is more effective in orienting the macromolecules along the extrusion direction. Additionally, this approach can be easily applied to extruded items, such as hoses and tubular parts [14,211,212,213].

**Figure 11:**
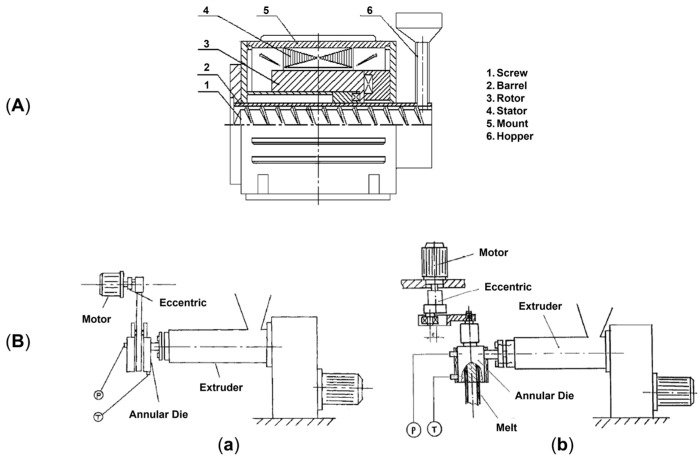
Schematic representation of the instrumental setup of vibration extrusion via (**A**) screw vibration [228] (adapted with permission from Taylor & Francis Ltd., 2007, http://www.tandfonline.com (accessed on 6 May 2025) and (**B**) oscillation die approaches. Inserts (**a**) [231] (adapted with permission from John Wiley and Sons, 2004) and (**b**) [229] (adapted with permission from Elsevier Science Ltd., 1990) illustrate the longitudinal and transverse application methods in the oscillation die technique.

Different approaches can be exploited for the actual application of an external field on a material. For instance, in oscillation extrusion it can be the screw vibrating, thus providing an overall plasticizing effect that improves the melting capacity and lowers the energy consumption of the extrusion process [206,207,219,220,227,228]. Also, in this case the application of the vibration is less localized, and the time of application is longer with respect to the alternative represented by the oscillation of the die [219]. In this latter case, a vibrating annular die characterized by an internal vibrating cylinder is exploited. This is the most used approach also owing to the possibility of applying the field alternatively in the longitudinal and transversal directions [206,210,229]. On the other hand, the vibrating die is the only possible approach when the vibration is applied in the direction parallel to the melt flow [210,214,223,230]. Schematic diagrams of the instrumental setups of the different approaches are shown in Figure 11. In particular, Figure 11A represents the screw vibrating approach, while Figure 11B (inserts a and b) illustrates the longitudinal and transverse configurations used for the oscillation die method.

Focusing on the materials, in the present section the effect of the variation in terms of the frequency, amplitude and intensity of the vibration on the morphology and final properties of pristine polymers and blends will be discussed. It is worth noting that, if not differently indicated, the studies reported refer to extrusion with a vibrating die.

**Figure 12:**
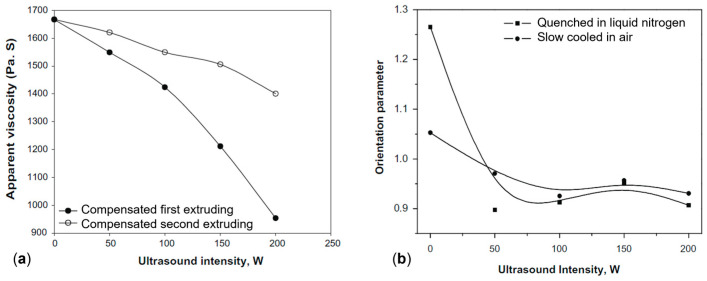
(**a**) Amended apparent viscosity of the PP after the first ultrasonic vibration-assisted extrusion performed at different intensities and the corresponding material reprocessed with no vibration applied. (**b**) Relation between the orientation parameter and the ultrasonic vibration intensity after the first processing for the materials quenched or slow-cooled in air [210]. Adapted with permission from Elsevier Science Ltd., 2010.

In the study of Chen at al. [210], PP was processed with an ultrasonic vibration at 20 kHz, at intensities of 0, 50, 100, 150 or 200 W. In order to differentiate between physical and chemical contributions, two subsequent extrusions were performed: the first one with the application of an ultrasonic field at the selected intensities and the second one without it. Figure 12a shows the variation of the apparent viscosity measured after each extrusion step. It is worth noting that the reported viscosity values have been amended in order to take into consideration the viscosity reduction induced by the extrusion itself. For the materials after the first processing step, the viscosity exhibits a monotonic decrease as a function of the applied intensity. This trend was attributed to both chemical and physical factors. In particular, the first one promoted chain-scission reactions leading to a decrease in the molecular weight and hence the viscosity. Otherwise, the physical effect is responsible for improvement in the macromolecular mobility and a decrease in the elastic tensile strains, both contributing to decreasing the material viscosity. As is observable from Figure 12a, also during the second extrusion step, the PP viscosity continues to decrease as a function of the intensity applied during the first extrusion step, despite this drop being less significant compared to that recorded in the first processing. Since no ultrasound was applied, the recorded viscosity reduction can be ascribed to the chemical effect undergone by PP during the first extrusion.

Furthermore, the effect of the application of the external field on the orientation of the macromolecules was investigated (Figure 12b). It was found that the chain orientation decreases with increasing vibration intensity up to an average value. This phenomenon was related to the enhancement in the motion of the macromolecules due to the energy provided by the vibrations, given that lower relaxation times are obtained when the number of entanglements and interactions decrease [210,217]. Thus, an overall more disordered morphology is expected when a high-intensity ultrasonic field is applied.

Similarly, Guo et al. [214] documented a decrease in the apparent viscosity with the increase in the intensities of ultrasound vibration (namely, 0, 50, 100, 150, 200 and 250 W) during the ultrasonic extrusion of linear low-density polyethylene (LLDPE). Additionally, a linear relationship between the apparent viscosity and the applied shear rate was detected in the range of 30–100 s^−1^ for all the intensities of ultrasound considered. Also, it emerged that the sensitivity of the apparent viscosity from the shear was related to the vibration intensity. In fact, with the increase in the shear rate, the greater decrease in the apparent viscosity value was observed for the material processed at a higher ultrasound intensity.

The dependence of the apparent viscosity sensitivity on the values of some processing parameters was observed also by Gao et al. [221] for the oscillation extrusion of HDPE, exploiting frequencies between 0 and 93.3 Hz and different values for the die temperature and screw rotation speed.

The obtained results showed that the apparent viscosity decreases from 950 to 600 Pa.s passing from 0 to 9.3 Hz. Then, it increases again, reaching 700 Pa.s at 20 Hz, maintaining a quite constant trend afterwards. Therefore, the greater sensitivity is reached at a low vibration frequency, while the contribution is almost negligible at high frequencies. Additionally, it was demonstrated that this trend is only observed at a low screw rotation speed, while for higher values the impact of the vibration frequency on the final apparent viscosity is almost negligible.

Lastly, the resulting mechanical properties were analyzed in relation to the vibration frequency. In particular, it appeared that the longitudinal yield strength of the extruded materials abruptly increases from 21.31 MPa in the case of conventional extrusion to over 23 MPa as soon as vibration is applied. Also, a similar behavior was observed in the transverse direction. While in the longitudinal direction this result was expected considering the disentangling and orienting effect of the vibration field on the macromolecules, it was not obvious in the case of the transversal one. The observed behavior was explained considering both the morphological and thermal results for the materials processed at 0, 5.6 and 9.3 Hz. What emerged is the increase in the overall anisotropy in the presence of a higher vibration intensity, thus resulting in a better orientation of the crystals in the longitudinal direction. On the other hand, this resulted in a slight increase in the final crystallinity and in the formation of smaller crystals characterized by a lower lamellar thickness in the case of the materials processed at 5.6 and 9.3 Hz when compared to those obtained at 0 Hz. As a consequence, the improvement in the mechanical properties in the transversal direction owing to the refinement of the crystals was greater than the loss related to their orientation along the longitudinal axis. Thus, the study assessed the action of the biaxial reinforcement effect resulting from the application of oscillation.

**Figure 13:**
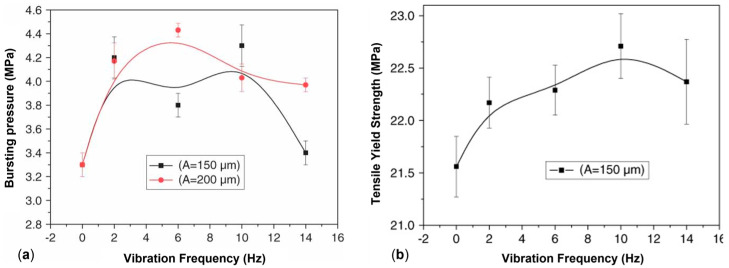
(**a**) Behavior of the bursting pressure depending on the vibration frequency. (**b**) Trend of the yield strength in the MD as a function of the vibration frequency. In both cases, “A” corresponds to the adopted amplitude [219]. Adapted with permission from John Wiley and Sons, 2009.

A beneficial effect of the applied vibration on the mechanical properties was also observed by Kaiyuan et al. [219], who exploited an oscillation extrusion and screw vibration approach in the frequency range of 2–14 Hz and a vibration amplitude of 150 or 200 μm. As shown in Figure 13a, for the bursting pressure a non-linear behavior was obtained at both of the frequency amplitudes. In fact, the value jumped from 3.3 MPa at 0 Hz to about 4.2 MPa in the range of 2–10 Hz. On the other hand, while this value was maintained also for higher frequencies in the case of the highest vibration amplitude, it decreased for the processing carried out at 150 μm. Nonetheless, in all cases the final value obtained in the presence of vibrations is higher than that reached in conventional extrusion, proving the beneficial effect of the application of the external field on the performances in the circumferential direction of the pipes. On top of that, the mechanical properties in the machine direction are also maintained or improved (Figure 13b). Thus, the positive effect of the presence of the vibration emerges in both the longitudinal and transversal directions of the pipe.

Additionally, from the thermal analysis emerged the improvement in the crystallinity content and perfection of the crystals with the enhancement of the frequency or amplitude of the oscillation [219]. In particular, the overall crystallinity increased from 57.61% at 0 Hz and 150 μm to 61.26% at 14 Hz, while the melting temperature improved from 133.7 °C at 0 Hz and 200 μm to 135.6 °C at 6 Hz. Such enhancements have been explained considering the disentangling effect of the vibration on the chains, which allows nucleation events at higher temperatures, causing the formation of more ordered and thermally stable crystals [68,219,221,232]. In addition, the simultaneous lowering of the entanglement density and the presence of a multiplex shear force, resulting from the simultaneous presence of the screw rotation and axial vibration, promotes the chain orientation in both the transversal and longitudinal directions with a consequent enhancement of the mechanical properties [208,219,221]. Such considerations are in accordance with other studies [222,228]. In particular, Qu et al. [228] evaluated the tensile strength in the machine and transverse directions of isotactic PP films produced with vibrations in the range of 0–14 Hz at an amplitude of 80 μm. What emerged was the enhancement of the values in both directions with the increase in the frequency up to 6 Hz and the further lowering of the property afterwards. Additionally, notwithstanding the common trend of the tensile strength as a function of frequency for both the MD and transverse direction, the decrease observed at high frequencies was more severe in the MD.

**Figure 14:**
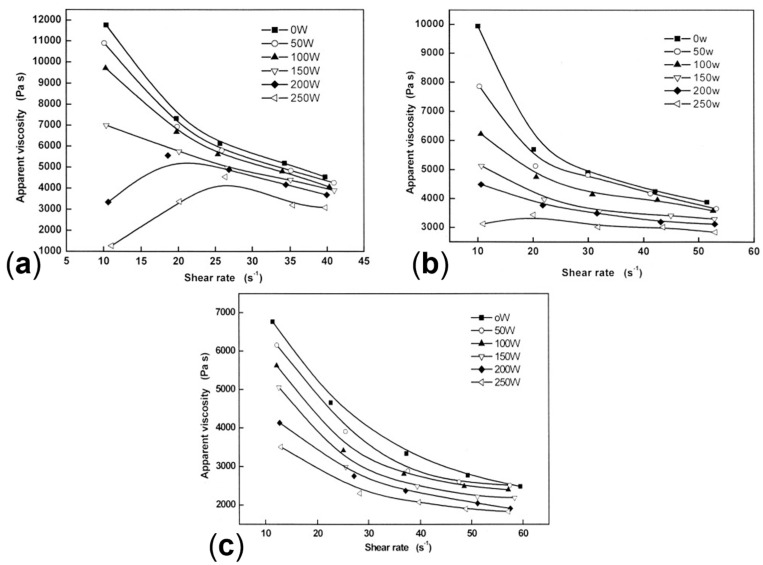
Variation in the apparent viscosity under different ultrasonic intensities for UHMWPE-PP blends containing (**a**) 10 wt%, (**b**) 20 wt% and (**c**) 30 wt% of PP [216]. Adapted with permission from John Wiley and Sons, 2003.

Moving forward to polymer blends, Liu et al. [216] analyzed the effect of the vibration intensity in ultrasonic extrusion of UHMWPE-PP blends containing 10, 20 or 30 wt% of PP. According to what has been reported so far, the apparent viscosity decreases with the increase in the frequency intensity (Figure 14). However, a different rheological behavior was observed depending on the PP content. In particular, the sensitivity from the shear rate lowered with the increase in the minor phase amount.

Additionally, a peculiar trend was observed in the comparison of the apparent viscosity as a function of the applied shear rate for the blends extruded at 200 and 250 W. In particular, as shown in Figure 14a, a positive slope characterizes the curves of the material containing 10 wt% of PP up to about 25 s^−1^. However, such a trend lowers with the increase in the PP content to 20 wt% (Figure 14b), and the shear-thinning behavior at a low shear rate can be appreciated for the blend in which the 30 wt% is present (Figure 14c).

In the study, this behavior was related to the UHMWPE. Specifically, the greater sensitivity of the blends containing a higher concentration of the latter is larger at a low shear rate due to the long exposure time of the long chains under high-intensity irradiation. As a result, a greater number of entanglements disappear but, at the same time, the macromolecules may face heavier breakage. Both phenomena are accentuated with a higher UHMWPE content and irradiation intensity. In fact, with the lowering of the exposure time resulting at a higher shear rate, the disentangling effect is reduced and the apparent viscosity increases.

**Figure 15:**
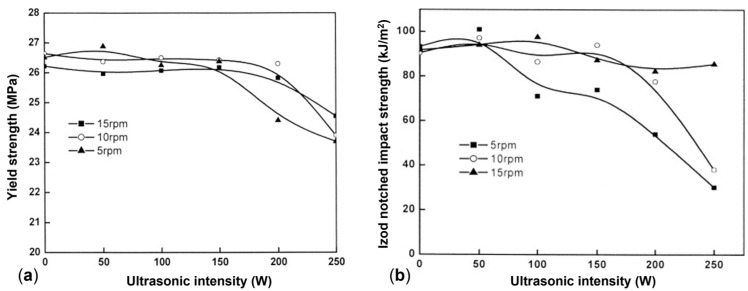
Mechanical properties of the UHMWPE-PP blend containing 20 wt% of PP ultrasound extruded at 5, 10 or 15 rpm. The results of the (**a**) yield strength and (**b**) Izod notched impact strength are reported [216]. Adapted with permission from John Wiley and Sons, 2003.

On the other hand, in the same study [216], a minor or detrimental impact of the ultrasonic field on the mechanical properties was observed at different screw rotation speeds (namely, 5, 10 and 15 rpm). For instance, in the blend containing 20 wt% of PP the yield strength was constant up to 150 W when processed at 10 and 15 rpm (Figure 15a). Then, the values decreased at 250 W despite the rotation speed considered.

An even worse situation is appreciated considering the Izod notched impact strength (Figure 15b). In fact, the value remains constant only in the case of 15 rpm, while it decreases from about 90 kJ/m^2^ to less than 40 kJ/m^2^ when the extrusion is carried out at 5 or 10 rpm. In this case, the lowering of the mechanical properties is related to the damaging effect of a too high ultrasonic intensity or too long irradiation time.

Interesting results on the increase in blend compatibility owing to the presence of an ultrasonic field were reported in [224,226]. Chen et al. [224] calculated the interfacial tension (α) and the volume-average particle radius (R) of HDPE–PS blends containing 20 wt% of polystyrene, exploiting the emulsion-type model proposed by Palierne [233]. The materials were compounded with or without the application of an ultrasonic field and a single (20 kHz and 200 W) or double (20 kHz and 100 W each) processing step was exploited. Then, the rheological behavior in terms of storage and loss moduli was evaluated through tests performed with a parallel-plate rheometer. The experimental values were compared with the ones calculated with the Palierne model, and the ones that fitted the best were selected. Afterwards, the Palierne relation was exploited to calculate the interfacial tension and the volume-average particle radius of the dispersed phase from the chosen calculated moduli. What emerged in the case of the single compounding step was the reduction in both α and R when the vibration-assisted process was considered. In particular, the first parameter lowered from 4.5 to 3.9 mN/m, while the second decreased from 1.14 to 0.83 μm. The reductions were representative of the increase in the compatibility between the HDPE and PS promoted by the application of the ultrasonic field.

Similar results were obtained in the case of double-step melt blending, where the interfacial tension and volume-average particle radius decreased from 5.2 to 1.53 mN/m and from 1.31 to 0.73 μm, respectively, in the presence of vibration during extrusion. Thus, the positive impact of the ultrasonic field on the improvement in the compatibility in HDPE—PS blends was assessed both in single- and double-step compounding. Also, from the comparison of the α and R values calculated for the materials processed twice or with a singular melt blending, the importance of the intensity of the vibration can be appreciated. Focusing on the interfacial tension, the value obtained from the two-step blend without an ultrasonic field (5.2 mN/m) was greater than the ones for both the materials processed in a single step (4.5 and 3.9 mN/m for 0 and 200 W). Thus, the double processing itself appears to be detrimental. On the other hand, when the vibration is introduced, the final α appears to be the lowest (1.53 mN/m), even if the vibration intensity is half the one exploited for the single compounding. That is, a double extrusion at 100 W is more effective in promoting the compatibility between the polymers than a single extrusion at 200 W.

**Figure 16:**
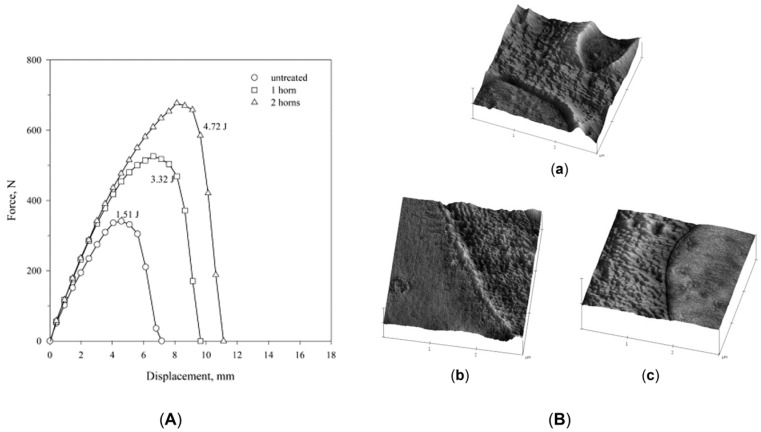
(**A**) Force–displacement dependence for the conventionally extruded and ultrasonic vibration-assisted blends resulting from the probe impact test. The energy impact calculated as the integral of the area below the curve is reported next to each graph. (**B**) Three-dimensional AFM surface profile PP\NR blends processed (**a**) without vibration or treated with (**b**) one horn or (**c**) two horns [226]. Adapted with permission from Elsevier Science Ltd., 2003.

Similarly, improvement in compatibility was reported by Oh et al. [226] based on the stress–strain curves for a 50 wt% PP–50 wt% natural rubber (NR) blend produced with ultrasonic vibration at an amplitude of 10 μm. In particular, the maximum strain increased from 9 to 12 MPa, while the strain at break increased from about 25% to over 120%. On the other hand, the study found a great improvement in the impact resistance, evaluated with the probe penetration test. As shown in Figure 16A, both the force and displacement enhanced. Thus, the impact energy increased from 1.51 J for the material extruded in the absence of vibration up to 3.32 and 4.72 J when the blend was ultrasonically compounded with one or two horns, respectively. Such improvement was related to the presence of an interfacial transition layer between PP and NR, as emerged from the AFM analysis. The three-dimensional surface profiles of the blends processed in the absence of vibration and with one or two horns are shown in Figure 16B (inserts a, b and c). As can be appreciated, in the first case the interfaces between the two materials are represented by sharp steps, which indicated a poor adhesion between the two, while a more irregular transition zone also affected by roughness characterizes the interface between the polymers processed in the presence of an ultrasonic filed. This was observed when one or two horns were exploited. Such a peculiar rough topology indicates the good interfacial adhesion reached in the materials treated with vibration; thus, the compatibility between PP and NR is promoted.

The evidence of in situ copolymerization in immiscible blends due to the presence of ultrasonic vibration was also reported by Isayev et al. [225]. In their work, PP or HDPE were alternatively blended with NR, ethylene-propylene-diene rubber (EPDM) or styrene-butadiene rubber (SBR) with a relative concentration of 50 wt%. The plastic–rubber blends were produced with conventional extrusion or in the presence of ultrasonic oscillation with a frequency of 20 kHz, a power of 3 kW and an amplitude of 6 or 10 μm. Also, two horns were exploited.

From the mechanical characterization the positive impact of the presence of the vibration emerged. In particular, the values of the elongation at break and Young’s modulus increased from 38.9 up to 126.8% and from 191 to a maximum of 250 MPa when considering the untreated or ultrasonic-assisted extruded PP\NR, respectively. Also, for HDPE\NR, the maximum elongation at break was 300.6%, considering an untreated value of 189.0%, while the Young’s modulus increased from 75.1 to 127.0 MPa. On the other hand, in the presence of EPDM the improvement was more contained. Specifically, for PP\EFDM the elongation at break and Young’s modulus changed from 29.1 to 121.2% and from 229.0 to 278.0 MPa, respectively. Lastly, for HDPE\EPDM the two increased from 88.2 to 147.8% and from 103.0 to 125.0 MPa.

Additionally, considering the impact energy with the probe test, an increase between 1 and 2.5 J was observed in all cases, and the best improvements were reached in the presence of NR, namely, from 2.75 to 5.89 J in PP\NR and from 6.09 to 8.12 J in HDPE\NR.

It is worth noting that the changes in the mechanical performances due to the different frequency amplitudes were secondary considering that the most important impact resulted from the presence of the vibration. Such results were explained considering the predominant role of the chemical effect on the reduction in the apparent viscosity of the treated materials, which was also assessed. In fact, the rupture of the macromolecules would provide long-chain radicals from both polymers in the blend, which tend to recombine, resulting in the formation of the copolymer at the interface. Ultimately, this would promote the adhesion of the two and promote an overall better dispersion.

In addition, the formation of the copolymer was further confirmed from the comparison of the SEM micrographs of the pre- and post-annealing blends. The annealing treatment lasted 10 min and was performed at 160 °C for the HDPE-based and 190 °C for the PP-based blends. What emerged was the increase in the dispersed-phase domain size only in the conventionally extruded material, while the small dimensions were retained in the treated ones. Such observations were related to the presence of the copolymer at the interface for the blends processed in the presence of a vibration field, which limited the growth.

### 2.4. Drawing

A further approach allowing tailoring of the microstructure of polymer-based systems exploits the key role of the elongational flow owing to the application of drawing aligned to the extrusion direction. In fact, the superior ability of the velocity gradient parallel to the material flow when compared to the perpendicular one in promoting the polymer structuring in terms of macromolecular orientation, amorphous to crystalline balance, and breakup and dispersion of the minor phase in polymer blends has been assessed over the years [5,17,114,161,234]. Additionally, the increase in the elongational flow intensity determines the microstructure transitions from a homogeneous organization of spherulites to an anisotropic distribution of deformed spherulites and small lamellae to a dispersion of well-oriented lamellar structures [5,142,235]. Such versatility in shaping the microstructure results in good potentialities for defining tunable final properties. As a consequence, several production processes exploiting elongational flow have been developed, including film casting, film blowing and melt spinning [5,17,114,143,236]. In addition, such techniques are extensively exploited at the industrial scale for the production of packaging [90,237,238] and fishing nets [142,144] and in biomedical [91,145,147,239,240] and textile applications [91,143,148] owing to the good combination of high-speed production, the cheapness of the final products, the high mechanical properties and the good barrier performances. On the other hand, the present discussion will focus on film casting and melt spinning in view of the greater efficiency and uniformity in the thickness of the film produced with the former technique when compared to the ones obtained via film blowing [241] and to the high quantity of filament ensured by the latter [143]. Additionally, film casting and melt spinning share the need of a high supercooling and/or a strong flow field in order to promote the increase in the anisotropy of the material [5].

#### 2.4.1. Film Casting

Firstly, in film casting the machinery is equipped with a center-fed “T” die and the elongational flow is applied during solidification in non-isothermal conditions of the material in the air gap right after the extruder. The drawing is performed by water-chilled or heated rollers in order to maintain a constant temperature and ensure complete cooling [90,241]. Alternatively, a precursor film may be produced first and the actual stretching can be applied afterwards [5,242,243]. Lastly, an annealing step may be present [244,245].

The most important parameters for the determination of the microstructure are the draw ratio (DR) and the roll temperature [11,18,246,247,248,249,250,251,252,253,254], where the DR in films corresponds to the ratio between the rotation speed of the drawing rollers and the extrusion velocity [255].

##### The Role of the Draw Ratio

**Figure 17:**
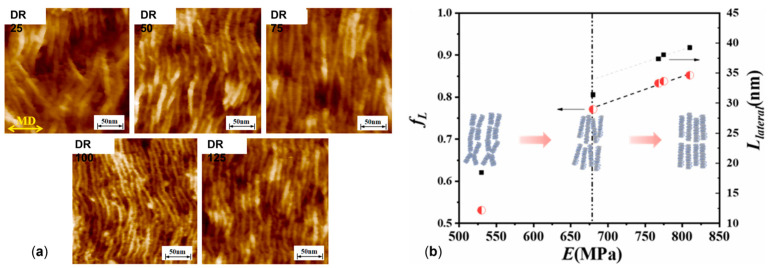
(**a**) AFM images of the film casted PBS at different DRs. (**b**) Lamellar orientation (f_L_) and lateral size (L_lateral_) expressed as a function of the elastic modulus [247]. Adapted with permission from Elsevier Science Ltd., 2021.

Firstly, considering the DR, its effect in determining the orientation of the macromolecules, the overall crystallinity content, the morphology of the crystals and the crystalline size was studied [5,37,114,149,247,248,249,251,256,257,258,259,260]. The major impact refers to the enhancement of the chain orientation in the direction parallel to the stretching, thus promoting the formation of oriented structures and increasing the overall anisotropy [247,252,253,256,258]. This phenomenon can be clearly appreciated in the work of Xie et al. [247], in which poly(butylene succinate) (PBS) films were obtained at DRs of 25, 50, 75, 100 or 125. In Figure 17a, the microstructures obtained in the different processing conditions are reported. What emerges is that at DR 25 the microstructure is characterized by deformed spherulites. Then, the morphology progressively evolves toward the formation of raw-nucleated oriented lamellar structures up to a draw ratio of 75. Such an improvement in the overall orientation was confirmed by the enhancement of the orientation degree, which improved from 0.46 to 0.73 when the DR was 25 or 50, respectively, while it was quantified at 0.80 with a draw ratio of 125. Thus, on the one hand, the great impact of the DR on the orientation was evidenced but, at the same time, it was assessed that a larger transformation of the microstructure is obtained in the low DR value range. Additionally, the role of the draw ratio in the formation of a specific polymorphic phase was investigated. In particular, considering the presence of the α and β crystalline phases in PBS, the suppression of the latter with the increase in the applied DR emerged. The phenomenon was related to the formation of a more oriented structure with the enhancement of the draw ratio, promoting a stable α phase. Additionally, the increase in the chain orientation was also identified to be the basis of the larger lateral dimensions of the lamellae obtained at higher DRs. Conversely, no relation was appreciated between the DR and the crystallinity content. In fact, the value was quite constant independent of the draw ratio considered. In this case, the authors suggested that this was related to both the low crystallization rate affecting PBS and the short time spent by the material between the die and the rolls in which the drawing actually occurred [247].

As a consequence of the variation in the microstructure, the tensile properties changed. Specifically, both the tensile stress and elastic modulus improved with the increase in the applied DR. Additionally, a linear relationship emerged to relate the elastic modulus with the lamellar orientation at a DR greater than 50, when spherulites are no longer present in the microstructure (Figure 17b).

Focusing on the overall crystallinity, ambiguous evidence is found in the literature when analyzing the relationship with the DR. Specifically, some sources report the negligible effect of the draw ratio [247,256], while others refer to the increase in the crystallinity content with the applied stretching, eventually followed by the abrupt decrease in the value at high DR values [149,252,253,260,261,262]. This divergence may be related to the interplay between the crystallization kinetic of the polymer considered and the duration of the time interval in which the drawing is applied before complete solidification. Specifically, taking into consideration the speed at which film casting is performed, if the crystalline formation is slow, the presence of the stretching has no effect on the crystallinity content because not enough time is provided for the crystallization to occur [253]. However, if the solidification rate is adequately low, the drawing has a positive effect in terms of improving the crystallinity content because the formation of crystals is favored. Additionally, less defective lamellae are obtained owing to the orientation of the macromolecules promoted by the presence of the elongational flow. On the other hand, with the increase in the DR, the system will reach a configuration in which the solidification rate is no longer adequate for the crystallization kinetic. That is, the high drawing speed results in the formation of heavily defected lamellae that are no longer able to form ordinated crystalline structures. Thus, the overall crystallinity decreases [252,253]. It is worth noting that it is not possible to identify a unique DR value after which the crystallinity content decreases because this is strongly related to the polymer considered [149,252,253,262].

**Figure 18:**
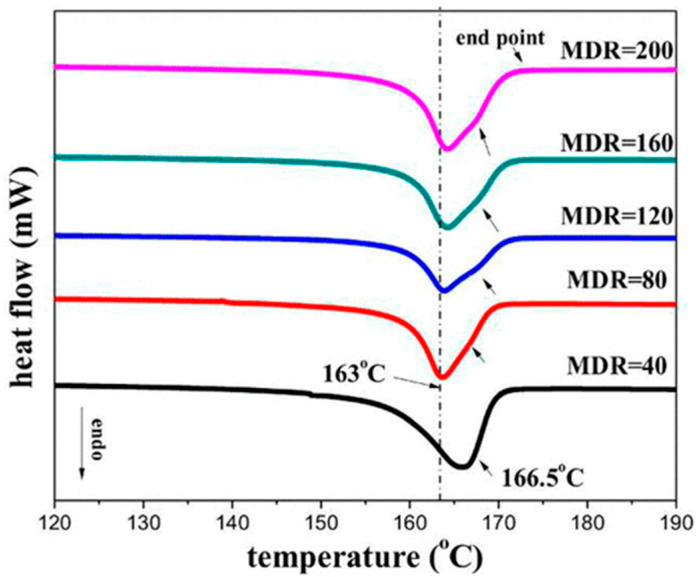
DSC analyses of PP films drawn at different DRs [256]. Reproduced with permission from the American Chemical Society, 2015.

In addition, it is important to distinguish the effect of the DR on the overall crystallinity content from the one on the orientation of the crystals. The latter is always observed with an increase in the DR [149,247,248,252,262,263,264]. A clear example is provided by the study of Xu et al. [256] on PP films obtained with draw ratios of 40, 80, 120, 160 or 200. In Figure 18, the corresponding heating ramps of differential scanning calorimetry (DSC) are shown. Firstly, the crystallinity content was evaluated and for all samples was about 48%. Conversely, the clear differences in term of melting temperatures and the shape of the curves allowed two regions of evolution to be distinguished in the microstructure depending on the draw ratio applied. The first region corresponds to DRs ranging from 40 to 120, in which the lowering of the melting temperature testifies to the evolution of the shape of the spherulites. Specifically, with the increase in the DR, the circular spherulites become ellipsoidal and smaller in their dimensions. Then, raw-ordered lamellae are progressively formed at the expense of the ellipsoidal structures, up to the point at which a homogenous distribution of oriented lamellar domains is achieved. This evolution of the microstructure is driven by the intensity of the elongational flow applied, which increases the crystallization rate, which, in the end, exceeds the mean relaxation time of the chains [242,254,256]. Additionally, the second region was identified above DR 120, in which the melting temperature is no longer affected by the increase in the draw ratio, while the temperature of the end point of the peak increases along with the intensity of the shoulder in the high-temperature part of the peak. These two characteristics indicates the refinement of the oriented lamellar domains with the formation of more perfect and larger crystals and, eventually, of shish kebabs [245,256].

**Figure 19:**
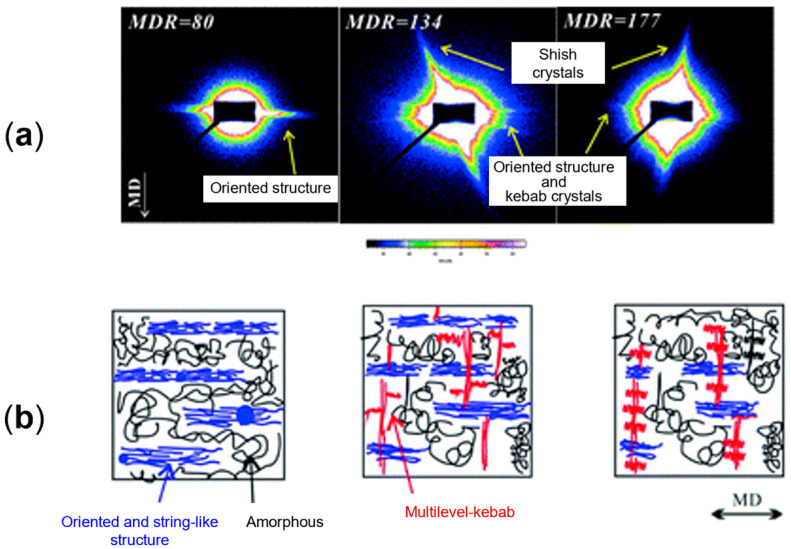
(**a**) SAXS spectra of samples extruded at different DRs and (**b**) corresponding schematic representations of the macromolecular organizations (the white arrow indicates the drawing direction) [253]. Adapted under CC BY 4.0 license.

As mentioned above, the enhancing of the orientation resulting from the increase in the DR may ultimately promote the formation of shish kebabs [245,252,253,256,265]. For instance, this was observed by Xu et al. [253] in PLA films produced at DRs of 80, 134 and 177. In Figure 19a the small-angle X-ray scattering (SAXS) spectra of the samples are shown. The X-ray analysis of the PLA film produced at DR 80 testifies to the increase in the anisotropy peculiar to the application of a low DR. In particular, the presence of oriented lamellar structures in the sample was detected. Additionally, with the increase in the DR to 134, the presence of shishes aligned in the direction of application of the drawing is clearly evidenced. Also, a similar morphology was detected when the draw ratio corresponded to 177. However, in the latter condition the verticality of the signal relative to the shishes indicates the improvement in their alignment with the direction of application of the elongational flow. In addition, the formation of kebabs was evidenced by the scattering of the pattern in the equatorial direction at DR 177. In fact, this signal was the result of the superposition of the oriented lamellae relative to the kebabs formed on the shishes. That is to say, a multilevel microstructure was obtained. A schematic representation of the microstructure in the three cases is shown below the SAXS image (Figure 19b).

**Figure 20:**
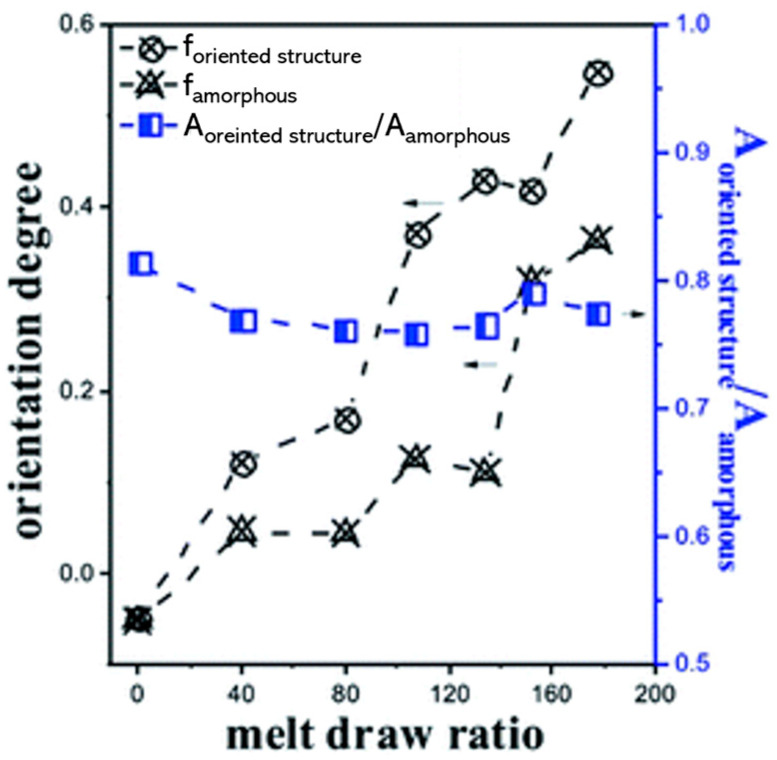
Orientation degree of the crystalline (f_oriented structure_) and amorphous (f_amorphous_) phases at different DRs and corresponding relative orientated contents of the two (A_oriented structure_/A_amorphous_) [253]. Reprinted under CC BY 4.0 license.

It is worth noting that the elongational flow has a strong impact not only on the orientation of the crystalline phase but also on the amorphous one, as clearly evidenced in Figure 20 [252,253,264,266]. In their study, Xu et al. [253] monitored the evolution of the orientation of the two phases with the increase in the DR, assessing a similar trend in the increase in the anisotropy in both of them. In fact, the relative orientated content of the two (A_oriented structure_/A_amorphous_) is quite constant with the increase in the DR. Additionally, the fact that the enhanced orientation in the amorphous phase does not promote the formation of new crystals is explained by the greater entanglement density hindering the mobility of the macromolecules along with the higher intermolecular cohesion in the crystalline phase, which requires a remarkable conformational ordering for the formation of a crystal. However, the increase in the orientation in the amorphous phase with the DR was also suggested to favor the reorganization of the amorphous chains into lamellar structures growing as kebabs around the oriented shishes [252].

**Figure 21:**
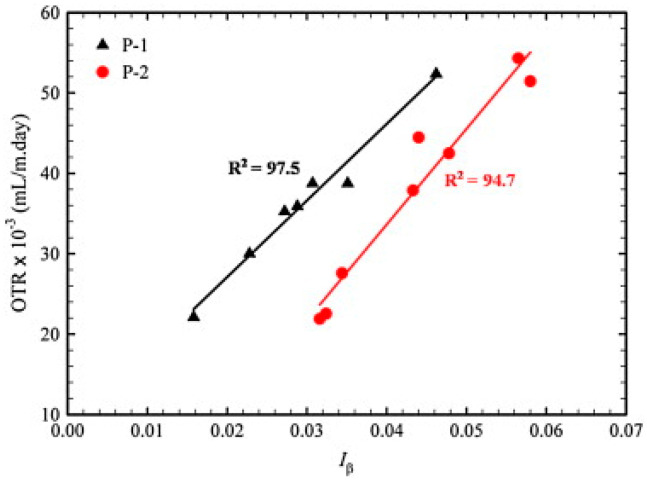
Oxygen transmission rate of the two films expressed as a function of the β-relaxation of the amorphous phase. The data for the precursor with only spherulites are in red, while the ones for the film with the co-existence of spherulites and lamellae are in black [264]. Reproduced with permission from Elsevier Science Ltd., 2009.

In addition, the impact of the draw ratio on the orientation of the amorphous phase has an impact on the mobility of the macromolecules, which ultimately affects the final characteristics of the films [253,264,266,267], for instance, barrier properties [264,267]. In fact, the gas permeability in semicrystalline polymers is related to the mobility of the chains in the amorphous region. That is, when the motion of the chains is hindered, better barrier properties are achieved. Thus, considering that higher DRs promote an increase in the rigid amorphous phase content, lower gas permeability is achieved. That was observed by Tabatabaei et al. [264] in PP films characterized by a different initial microstructure and drawn at DRs ranging between 1 and 7.2. In particular, one precursor presented only spherulites, while the second one had a co-existing morphology of spherulites and lamellae. This means that a more oriented microstructure characterized the latter. In Figure 21, the oxygen transmission rate (OTR) of the samples is expressed as a function of the mobility of the amorphous phase, which was monitored with the dynamic mechanical β-relaxation peak. From the data it emerges that in both cases the OTR decreases with the lowering of the mobility of the amorphous chains and thus with the increase in the DR. However, considering that the black data are representative of the films obtained from the more oriented precursor, it emerges that lower OTRs can be obtained with the same stretch extent if a greater starting orientation is present, and thus a final lower mobility of the amorphous phase is achieved.

##### The Role of Chilling-Roll Temperature

On top of that, the chilling-roll temperature has proved to be an important parameter for the determination of the microstructure in cast films. In fact, it is strongly related to the time provided for the arrangement of the macromolecules before complete solidification. Thus, this parameter affects not only the crystallinity content but also the polymorphic phase formed and the dimension of the lamellae [11,18,246,250].

**Figure 22:**
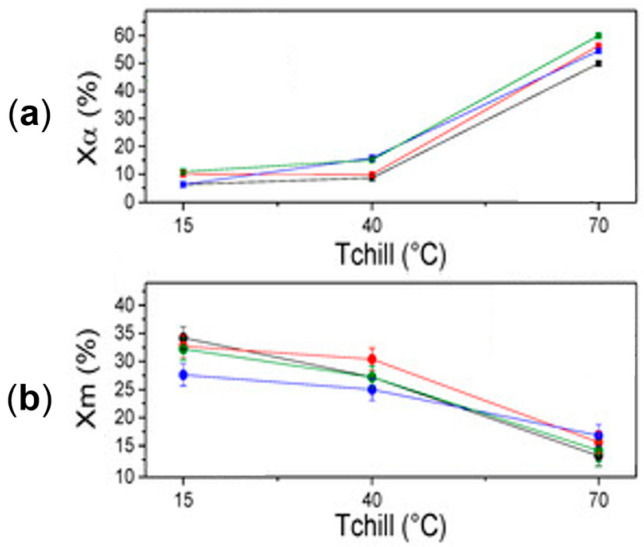
Crystalline content of the (**a**) α and (**b**) mesomorphic crystalline phases in PP films as a function of the chilling-roll temperature [11]. Reprinted under CC BY 4.0 license.

The effect of three different chilling-roll temperatures (15, 40 and 70 °C) on the final microstructure and properties of PP casted films can be clearly appreciated in the study of Di Sacco et al. [11]. The samples were obtained by exploiting different temperatures for the melt and throughput capacities, which had a minor role when compared to the chilling-role temperatures and, as a consequence, are not discussed in the present document. As shown in Figure 22a,b, it emerged that a lower quantity of the stable α phase and a greater content of the mesomorphic one is associated with a lower chilling-role temperature. This was related to the more rapid cooling induced by the roll temperature of 15 °C when compared to the one at 40 °C. As a consequence, in the first case a shorter time was provided for the organization of the chains, which was not enough to obtain the α phase. This evolution of the crystalline phases in PP film was in accordance with the observations of Liu et al. [250], which evidenced that the formation of the crystalline phases in PP may even be suppressed depending on the chilling-roll temperature. Specifically, under 40 °C only the mesophase was obtained, while solely α is present when temperatures greater than 80 °C were exploited.

In both studies, the evolution of the α over the mesophase content depending on the chilling-roll temperature was related to the resulting mechanical properties of the films [11,250]. What emerged was the increase in the tensile modulus in the machine direction with the enhancement of the α content, that is to say, when a greater chilling-roll temperature was used. On the other hand, Di Sacco et al. [11] assessed that the exploitation of a lower chilling-roll temperature is favorable for the lowering of the haze of the film. In fact, this property was related to the α-phase content because the crystalline phase represents an obstacle for the path of the light. The balance between the transparency, the ductility and the tensile strength is particularly important when the application as packaging is considered. Thus, the selection of the most appropriate chilling-roll temperature is strictly related to the final properties of interest.

Furthermore, the chilling-roll temperature emerged as having an impact also on the lamellar size [11,18]. In this case, the higher temperature promotes an increase in both the dimension and thickness due to the longer time allowed to the chains for their organization. Additionally, Tabatabei et al. [18], in their work on PP, assessed that the distribution of the crystalline size could be selected depending on the roll temperature. Specifically, for temperatures equal to or greater than 110 °C the formation of a bimodal size distribution was obtained, while at lower values a monomodal one was always observed. Such an evolution of the morphology was related to the longer time for relaxation provided to the chains at a higher chilling-roll temperature, which likely allowed the formation of lamellae by the low-molecular weight chains.

##### The Role of the Annealing Treatment

Lastly, the impact of annealing on the microstructure of casting films is considered. Such a step may be introduced in the production of cast films in order to lower the residual stresses and promote reorganization adjustments of the macromolecules [244,245,257,268,269,270,271,272]. In fact, it has been demonstrated that after a thermal treatment at an adequate temperature and for an adequate length of time, the crystallinity content in a film increases [252,263,271,272]. This phenomenon is due to the rearrangement of the amorphous chains in the proximity of the crystals, which organize according to the order already present [252,268]. Also, secondary crystallization is observed in the amorphous phase [244,245,268]. However, the formation of secondary lamellae requires the initial presence of crystals to coordinate the crystallization of the amorphous macromolecules [252]. In addition, the application of an external stress during the annealing process may further promote the overall anisotropy when compared to annealing performed without one [263]. On the other hand, it has to be taken into account that the temperature and time for the annealing process have to be properly selected depending on the polymer treated. In fact, if too high temperatures over too long times are adopted, an abrupt increase in the crystallinity content and a decrease in the orientation are obtained. Thus, the isotropy of the microstructure is promoted instead [263].

#### 2.4.2. Melt Spinning

Considering melt spinning, the molten material is compounded with an extruder and the filament is obtained owing to an ad hoc die, named a spinneret [143]. This particular die is characterized by a number of holes that can reach up to several hundred, allowing the production of a huge quantity of fibers simultaneously [143,273,274,275]. Additionally, a melt pump is often used to ensure a constant flow rate toward the die. After the spinneret, the material is concurrently cooled to achieve full solidification and stretched, thanks to a filament draw-down unit [143]. Lastly, after drawing, the filament is wound into a bobbin [143,274].

The drawing step may be performed online or offline, and the last approach is the most commonly used due to its higher compatibility with the industrial scale [143,273,274,275]. Also, in the case of melt spinning the stretching extent is monitored with the draw ratio, which, in this case, is defined as the ratio between the square value of the diameter of the extrudate divided by the square of the diameter of the fiber [276].

In the production of fibers, the most impactful parameters to be taken into account are the DR and the drawing temperature [142,150,151,235,277,278,279,280].

##### The Role of the Draw Ratio

**Figure 23:**
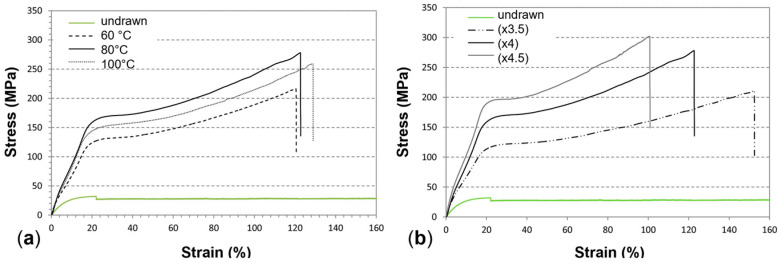
(**a**) Tensile behavior of the PBS fibers drawn at 80 °C at increasing DRs and (**b**) stretched at DR 4 at different temperatures [152]. Adapted with permission from Elsevier Science Ltd., 2019.

Considering the effect of the draw ratio, it has been extensively examined in the previous section regarding film casting. In the case of melt spinning, similar effects are appreciated. In fact, also in this case, the increase in the DR values is recognized to increase the crystalline and amorphous orientation and, thus, the anisotropy. On the other hand, the overall crystallinity content is always reported to increase with the increase in the draw ratio [150,151,235,277,278,279]. As a consequence, variation in the tensile properties is observed. In particular, as shown in Figure 23a, with the increase in the orientation and crystallinity resulting from the greater DR, progressive improvement in the tensile modulus and strength at break is achieved. At the same time, the lowering of the elongation at break is obtained [142,151,152,235]. However, it is worth noting that the increase in the tensile strength associated with the improvement in the DR is limited. In fact, the application of the elongational flow at the initial stages of the production of the filament promotes the orientation and crystallinity content; however, once the crystals are well-aligned along the stretching direction, the further increase in the draw ratio results in the damaging of the crystal structures. As a result, the maximum strength at break borne by the fiber decreases [142,150,281].

##### The Role of the Drawing Temperature

In addition to the DR, the drawing temperature represents an important parameter in the determination of the final microstructure and, thus, the final properties. In particular, it has an impact on the mobility of the macromolecules, affecting, for instance, the entanglement density in the amorphous phase [142,152,282]. As a consequence, a higher orientation in the drawing direction and an increase in the crystallinity content is obtained with the increase in the drawing temperature, thus promoting the overall anisotropy [152,277,282]. In fact, at lower temperatures, the predominance of the amorphous or mesomorphic phases over the crystalline one is often reported [154,155]. As a result, the tensile properties of the fibers drawn at higher temperatures show a stiffer behavior, characterized by a higher strength at break and elastic modulus, along with a lower elongation at break [155]. On the other hand, as can be appreciated in Figure 23b, the enhancement of the ductility may affect the mechanical properties of the fibers if the drawing temperature is too high [152]. This behavior is explained by the treatment temperature in comparison with the melting temperature of the polymer. Specifically, if the drawing temperature is close to the melting one, the relaxation and mobility of the macromolecules are promoted. Additionally, the slippage of the chains is favored by the presence of the drawing force [142,152,155,277], and, ultimately, the crystals may remelt [152,283]. As a consequence, the fibers bear a lower maximum strength.

### 2.5. Microfibrillation

The last microstructuring approach exploiting extrusion to be discussed is microfibrillation. This technique is specifically meant for immiscible blends, in which the formation of oriented polymeric fibrils of a second phase embedded in the matrix is obtained owing to the deformation of the solid state of the dispersed phase [1,20,284,285,286]. The main purpose is to increase the aspect ratio of the fibrils in order to enhance the contact area with the matrix and, thus, maximize the stress transfer from the matrix to the high-strength oriented structures. As a result, the microfibrillated blend is characterized by greater strength at break and a higher modulus when compared to the corresponding unstructured one [20,81,276,286,287,288,289]. In fact, to stress such sought improvement in the mechanical properties, the blends are often referred as microfibrillar composites (MFCs) [287,288,290,291], and the distribution, aspect ratio, orientation and shape of the fibrils can be tuned by properly selecting the polymers and the processing parameters [1,20,284,285,286].

Microfibrillation can be obtained with two different approaches, which are single-step microfibrillation and two-step microfibrillation. In the first case, the formation of the fibrils is achieved during compounding thanks to the solid-state deformation of the dispersed phase, driven by the shear and elongational flows developed inside the extruder during compounding [70,292,293]. On the other hand, the latter is the more traditional technique and is often referred as three-step microfibrillation. In this case, the polymers are firstly melted and blended, while the fibrils are formed afterwards, in the so-called *fibrillation step*, owing to the application of the elongational flow to the material. Lastly, a third step known as isotropization, consisting in the shaping into the final object, can be performed. This corresponds to a further thermal treatment performed with compression or injection molding and usually results in a decrease in the aspect ratio of the fibrils [1,54,287,288,294,295,296,297,298]. Due to the focus on the increase in the anisotropy, in the present document the isotropization step will not be explored further.

Lastly, regardless of the approach considered, it is worth noting that microfibrillation is a relatively versatile and cheap microstructuring technique because it exploits machinery commonly used for the transformation of polymeric materials [54,290,299,300,301].

#### 2.5.1. One-Step Fibrillation

##### Molten-State Approach Involving FIC

Two approaches have been proposed for one-step fibrillation during compounding. In the first case, both the matrix and dispersed phase are in the molten state, and the formation of the fibrillar structures is obtained owing to the simultaneous orientation and flow-induced crystallization occurring in correspondence to the slit die [301,302,303,304,305,306,307]. The geometry of the die is the capstone of the structuring achieved because it allows the application of intense flow fields and increases the residence time without drastically increasing the temperature [306]. Thus, the structuring can be performed even by exploiting a single-screw extruder [301,302,303,304,305,306]. Additionally, the fibrillation is retained after extrusion because, on the one hand, the material is cooled right after the die and, at the same time, the crystals formed owing to flow-induced crystallization are more ordered; thus, they are characterized by a higher melting temperature. As a consequence, even if the fibrils are retained at a high temperature before the complete solidification of the blend occurs, the possibility of them remelting decreases and the achieved aspect ratio is maintained [301,302,303,304,307].

**Figure 24:**
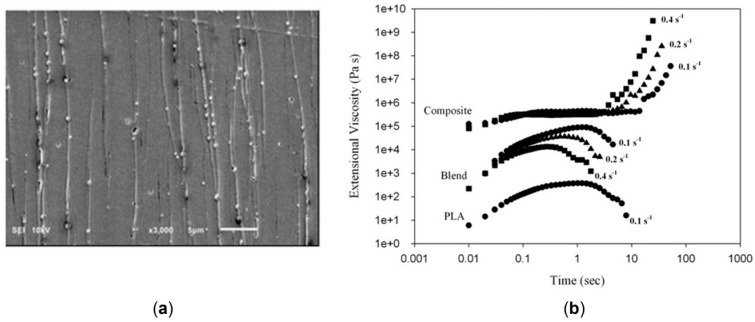
(**a**) SEM micrograph of the transverse direction of 97 wt% PLA\3 wt% PHA MFC. (**b**) Time-dependence of the tensile stress growth coefficient of the matrix (PLA), the compounded blend (Blend) and the MFC (Composite) at different Hencky strain rates [301]. Reprinted under CC BY 4.0 license.

A typical example of the fibrillar morphology obtained with this approach is reported by Vozniak et al. [301]. In their study, the fibrillation of 3 wt% of polyhydroxyalkanoate (PHA) in a PLA matrix was achieved, as can be appreciated in the micrograph of the sample in the transversal direction (Figure 24a). Additionally, in Figure 24b the time-dependence of the tensile stress growth coefficients of the matrix, the blend without fibrils and the MFC can be compared. In fact, in the latter case, the presence of a network of fibrils promotes strain hardening, which does not occur otherwise.

In addition, the presence of fibrils also has an impact on the mechanical properties because, owing to the high aspect ratio, an effective stress transmission from the matrix to the stiffer dispersed phase is promoted. For instance, in PLA\polybutylene adipateterephthalate (PBAT) MFCs the fibril network was claimed to be responsible for the increase in the elastic modulus and tensile strength without affecting the strain at break [303,305].

##### Solid-State Deformation Method

An alternative approach to obtain microfibrillation in one step was proposed by Jurczuk at al. [308] and is based on solid-state deformation. In particular, deformation of the crystals occurs when the stress transferred from the molten matrix to the crystal during compounding exceeds the critical shear stress of the slip plane of the crystal itself, and the effect is more effective when the system is characterized by a high capillary number. However, the dispersed polymer phase has to fulfill specific requirements in order for the fibrillation to occur. Firstly, the melting temperature has to be greater than the matrix one in order for a solid state to exist during compounding. Then, it has to be highly crystalline and characterized by a low density of entanglements to prevent premature breakage during deformation and, thus, to allow adequate deformation for the formation of the fibrils. As a consequence, typical second phases are nascent or as-polymerized UHMWPE and polytetrafluoroethylene (PTFE) [70,292,293,300,309,310,311,312].

The morphology of an MFC with this solid-state fibrillation during compounding can be modified by exploiting different screw speeds, processing temperatures and processing times, along with the relative content of matrix and dispersed phase [70,292,300,312]. In consideration of the first parameter, the increase in the screw speed corresponds to the enhancement of the shear rate which, on the other hand, results in a more intense viscous drag force and thus a greater capillary number. As a consequence, thinner and stronger fibrils are obtained at higher screw speeds [17,70,292,300].

**Figure 25:**
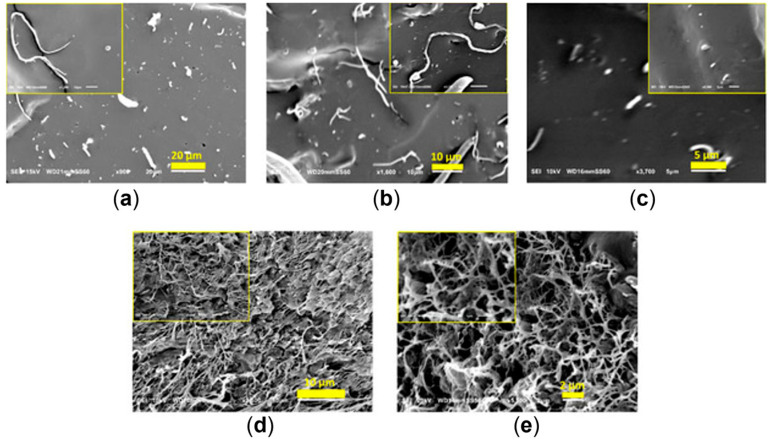
SEM micrographs of the polyolefin elastomer\UHMWPE blends compounded with a shear rate of 350 s^−1^ at different temperatures and processing times: (**a**) 75 °C for 10 min, (**b**) 75 °C for 30 min, (**c**) 75 °C for 90 min, (**d**) 115 °C for 10 min and (**e**) 115 °C for 30 min [300]. Reprinted under CC BY 4.0 license.

On the other hand, Hosseinnezhad et al. [300] studied the effect of the processing temperature and processing time on the fibrillation of 95 wt% polyolefin elastomer\5 wt% UHMWPE blends. The compounding was performed at a constant screw speed of 30 rpm, corresponding to a shear rate of 350 s^−1^. Temperatures of 75 or 115 °C were used, while processing times of 10, 30 or 90 min were exploited. In Figure 25, the SEM images of the blends processed in different conditions are reported. Firstly, from the comparison of the morphology obtained by varying the processing times, it emerges that the increase in this parameter promotes fibrillation. In fact, focusing on the material processed at 75 °C for 10 min (Figure 25a), the formation of fibrils with an aspect ratio of 70 was observed. However, when the processing time of 30 min was considered, the aspect ratio reached the value of 105 and the geometrical parameter further increased in the samples processed for 90 min (Figure 25c). Additionally, the positive impact of longer processing times on the aspect ratio was also confirmed in the samples processed at 115 °C, as can be appreciated from the micrographs after 10 and 30 min of compounding (Figure 25d,e, respectively).

In addition, in the study it was disclosed that the increase in the processing temperature positively affects the overall fibrillation. For instance, from the comparison of two samples processed for 30 min at 75 (Figure 25b) and 115 °C (Figure 25e), the extensive development of a fibrillar network clearly emerges in the second case. In fact, a more efficient fibrillation was observed with a higher processing temperature, allowing larger aspect ratios and a better distribution to be obtained.

Lastly, Jurczuk et al. [312] studied the rheological behavior of isotactic PP-, polystyrene (PS)-, HDPE- and low-density polyethylene (LDPE)-based blends in the presence of 1, 3, 5 or 7 wt% of native PTFE. What emerged is that a minimum content of 3 wt% of PTFE was required for the formation of the fibrils, independently of the matrix considered. Additionally, from the evaluation of the time-dependence of the tensile stress growth coefficient measured with uniaxial extensional deformation, strain hardening was appreciated in the MFCs containing at least 3 wt% of PTFE. In addition, the earlier onset of the strain hardening was associated with the larger content of PTFE in the blends [293,312]. Such rheological behavior is due to the presence of the network of fibrils of second phases and is particularly useful for the production of foams [70,293,310,311,312]. In fact, owing to the strain hardening, the cell concentration increases, the cell size is more stable and the coalescence lowers when compared to the foams obtained with the matrix alone [311,313,314,315].

The presence of the fibrils was also found to have a nucleating effect on the matrix, which is related to their content and aspect ratio. In particular, a well-structured network of fine fibrils represents a greater number of heterogeneous nucleating sites for the matrix when compared to low-aspect ratio domains of second phases. However, the network also represents a physical constraint for the growth of the matrix crystals, which are smaller than the ones obtained in the system with droplet-like domains of dispersed phases [292,309,311,313,316].

#### 2.5.2. Two-Step Fibrillation

In the first step, the two immiscible polymers are melted and blended in order to obtain a homogeneous dispersion of spherical domains of the second phase in the matrix. Then, the actual fibrillation step occurs owing to the application of a uniaxial elongation of the compounded material right after the die or at a later stage. In both cases, stretching is usually obtained owing to thermostatically controlled rollers, which also impart an orientation direction to the fibrils. Thus, morphological anisotropy is achieved [1,12,20,296]. In order for fibrillation to occur, the polymers have to fulfill some requirements. Firstly, a difference of at least 40 °C between the melting temperatures of the two polymers is required, and the high-melting temperature one has to be the second phase forming the fibrils. Additionally, the matrix has to be thermally stable in the range of processing temperatures typical of the dispersed phase. Lastly, the second phase has to be characterized by good elasticity, allowing the achievement of high aspect ratios in the fibrillation step [1,20,50,56,65,81,296,317,318,319].

It is important to stress that a fibrillated morphology is obtained owing to both shear flow and uniaxial elongation. That is, the first promotes the decrease in the second-phase domain size, improves their distribution in the matrix and promotes the deformation from spherical to ellipsoidal shapes. Additionally, the application of the drawing on such deformed domains culminates with the formation of the fibrils. Thus, the final highly oriented morphology is the result of the effect of both fields [1,320]. As a consequence, the final microstructure and, thus, the resulting properties are related to the processing parameters [1,20,57,296]. In the following, the role of the relative content of the matrix and dispersed phase, the viscosity ratio between the two, the presence of a compatibilized blend and the impact of different draw ratios on the structuring of immiscible blends will be discussed.

##### The Role of the Matrix-to-Second-Phase Content

**Figure 26:**
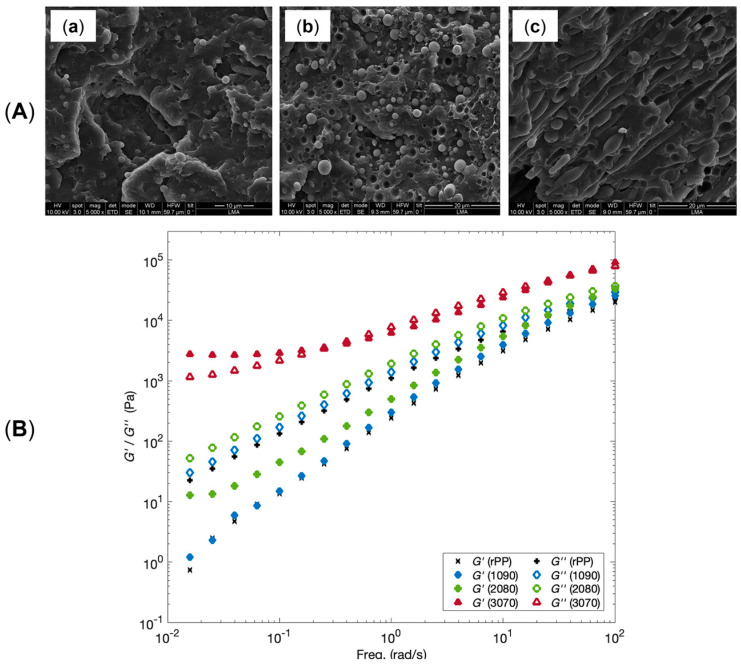
(**A**) SEM micrographs of the (**a**) 90:10 wt%, (**b**) 80:20 wt% and (**c**) 70:30 wt% PP\PET blends. (**B**) Storage (G′) and loss (G″) moduli as a function of the frequency measured for the neat PP and the three blends [322]. Adapted with permission from John Wiley and Sons, 2023.

Firstly, the relative content of matrix and second phase is discussed. This parameter is determinant for the achievement of the sea–island morphology during compounding, that is, the starting point for the formation of fibrils in the fibrillation step [57,289,317,321,322,323,324,325,326,327,328,329]. In the study of Kharghanian et al. [322], the microstructure was analyzed in PP\polyethylene terephthalate (PET) blends containing 10, 20 or 30 wt% of the latter. From the SEM micrograph of the 90 wt% PP\10 wt% PET, a sea–island morphology was observed (Figure 26A, insert a). However, the blend showed a liquid-like behavior similar to the one of the PP matrix (Figure 26B), which is not adequate for the formation of fibrils. Probably, sea–island domains could be appreciated also in the blend containing 20 wt% of PET (Figure 26A, insert b). However, the greater density of second-phase domains in association with the rheological behavior typical of a viscoelastic liquid (Figure 26B) is the desired condition for fibrillation to occur. On the other hand, the further increase in the PET content to 30 wt% was found to be detrimental to the formation of fibrils. In fact, as emerges from the SEM micrograph (Figure 26A, insert c), the dispersed phase forms a co-continuous path, and the rheological behavior of a viscoelastic solid can be observed at low frequencies (Figure 26B). Thus, in the latter case the high second-phase content does not create the proper conditions for fibrillation to occur, and the formulation with 80 wt% PP and 20 wt% PET was identified as the best option for MFCs.

**Figure 27:**
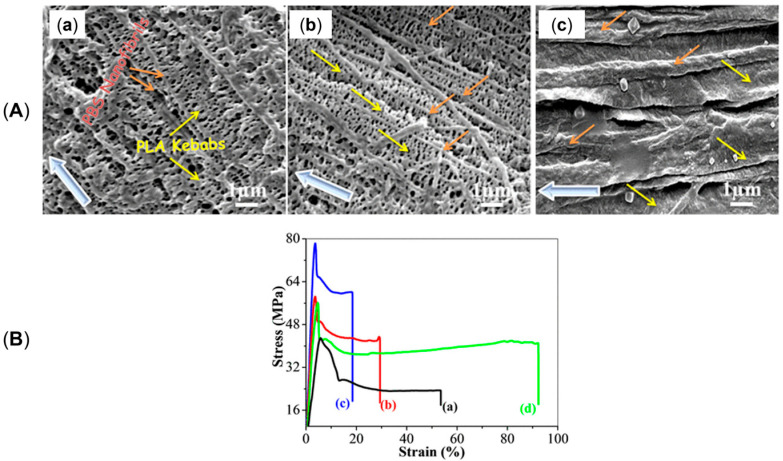
(**A**) SEM micrographs of (**a**) 90 wt%PLA\10 wt%PBS, (**b**) 80 wt%PLA\20 wt%PBS and (**c**) 60 wt%PLA\40 wt%PBS, in which the light blue arrows indicate the stretching direction, the orange arrows identify the PBS shish domains and the yellow ones highlight the lamellar kebab structures of PLA. (**B**) Stress–strain curves for (a) pure PLA, (b) 90 wt%PLA\ wt%10PBS, (c) 80 wt%PLA\20 wt%PBS and (d) 60 wt%PLA\40 wt%PBS [327]. Adapted with permission from the American Chemical Society, 2014.

In fact, other studies confirmed the above-described evolution of the morphology with the second-phase content [324,325,326,328,330]. Additionally, it was associated with the change in the mechanical behavior. For instance, Xie et al. [327] discussed the tensile properties of PLA-based blends containing 10, 20 or 40 wt% of PBS. As shown in Figure 27A, different combinations of yield strength and elongation at break were achieved. In particular, in all cases an increase in the yield strength was observed, but in the 90PLA\10PBS and 80PLA\20PBS blends this was associated with the lowering of the deformation at break. On the other hand, a larger deformation was obtained for 60PLA\40PBS. This behavior was described considering the different microstructures characterizing the samples (Figure 27B). In fact, even if in all cases the microfibrillation was appreciated, the morphology varied depending on the PBS content. As can be observed in Figure 27B, PBS forms shish-like structures oriented parallel to the stretching direction, which coordinate the formation of kebabs made of PLA lamellae, and thus shish kebabs are obtained. This microstructure provides self-reinforcement in the blend. However, a more ordered morphology is achieved in the presence of 20 wt% of PBS (Figure 27B, insert b) when compared to the one containing 10 wt% of second phase (Figure 27B, insert a), and the variation positively affects the mechanical performances with the enhancement of both the yield strength and elastic modulus. Nonetheless, the further increase in the second-phase content up to 40 wt% enlarges the diameter of the PBS domains, and, as a consequence, a peculiar combination of larger yield strength and deformation at break is achieved. In fact, the improvement in the strength and stiffness when compared to pure PLA is related to the oriented shish kebabs, while the ductility is obtained owing to the intrinsic flexibility of the PBS domains, which are larger when compared to the lower second-phase-content ones due to the collision and agglomeration occurring as a consequence of the high PBS content.

In fact, the increase in the diameter and diameter distribution of the microfibrils due to the greater second-phase content was confirmed by several studies [321,323,324,329], and the phenomenon of coalescence during the drawing step turned out to be crucial to explaining the development of the microstructure [50,321,323,329,331,332,333]. As explained by Huang et al. [321], in their work on PP\polyamide 66 (PA66) blends containing 5, 10, 15 or 20 wt% of PA66, the formation of fibrils was obtained independently of the second-phase content. However, the density of microfibrils increased at larger polyamide concentrations, while, considering a constant drawing ratio, the diameter decreased from 10 μm to 4–5 μm and the diameter distribution became narrower when the content of PA66 increased from 5 to 15 wt%. Nevertheless, the further growth of the PA66 content to 20 wt% promoted the rise in the average diameter up to 8 μm along with the widening of the corresponding diameter distribution. This behavior was explained considering the deformation, breakup and coalescence involving the second-phase domains during the drawing step [50,321,323,332,334]. In particular, the first two phenomena are related to the capillary number of the system, and, for low concentrations of the second phase, if the particles have an adequately large diameter, the dispersed domains are affected by deformation and eventually breakage due to the application of the elongational flow. On the contrary, the smaller particles retain their spherical shape. Thus, in the final morphology, both thin fibrils and small droplet-like domains of second phase are present. However, different transformations occur in the blend when the second-phase content reaches a value at which a high collision probability between the particles is obtained. In this case, the larger phase domains deform into fibrils due to the application of the drawing, while the smaller particles collide and coalesce due to the formation of droplets of a size large enough to undergo deformation as well. Additionally, the diameter at which the shape transformation occurs is determined by the capillary number of the system. As a consequence, in the final morphology, two populations of fibrils will be distinguished. Specifically, the first one is made of finer fibrils that originated from the initial droplets with an adequate diameter, while the second one is characterized by coarser fibrils resulting from the deformation of the collided and coalesced particles. Also, this explains why both the average diameter increases and the diameter distribution broadens. Thus, considering the results of Huang et al. [321], it can be concluded that the content of PA66 at which a good collision and coalescence probability was reached is between 15 wt% and 20 wt% because in the latter case the increase in the diameter of the fibrils was appreciated.

Further, the resulting mechanical properties reflected the evolution of the microstructure [321]. In fact, from the tensile tests emerged the enhancement of the tensile strength with the PA66 content up to 15 wt% and the decrease in the value afterwards. On the other hand, the elastic modulus increased until it reached a plateau for a second-phase content equal to or larger than 15 wt%. This behavior was explained by the superior strength of PA66 when compared to PP, along with the defects at the interface due to the poor interfacial quality. In fact, the mechanical strength initially increased owing to the second-phase fibrils. However, the voids formed at the interface during the tensile test hindered the stress transfer from the matrix to the dispersed phase and acted as stress concentration sites, thus lowering the tensile strength. At the same time, the elastic modulus was not affected by the presence of the voids because it is related to the number and aspect ratio of fibrils, both increasing with the PA66 content. Additionally, the plateau was reached because the density of the fibrils in the matrix was high enough that a further increase in their number provided a negligible effect.

On top of that, it was observed that coalescence depends not only on the second-phase concentration but also on the applied draw rate [323,332]. As reported by Gonzales-Nunez et al. [323] for HPDE\polyamide 6 (PA6) blends containing between 1 and 15 vol% of the latter, the deformation of the second phase was independent of its content when the process was performed at low draw rates. However, for higher drawing speeds, the same behavior was observed for second-phase concentrations ranging between 1 and 4 vol%, while for values greater than 5 vol% coalescence occurred with the increase in the content and/or draw rate. This was related to the enhanced collision probability, which promoted coalescence.

##### The Role of the Compatibilizer

**Figure 28:**
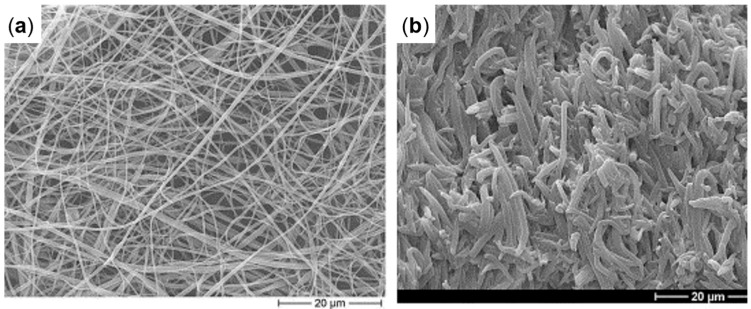
SEM micrographs of PP\PET blends (**a**) without compatibilizer or (**b**) with 1 wt% of additive [56]. Adapted with permission from Elsevier Science Ltd., 2005.

Secondly, an alternative mechanism to collision was proposed in order to explain coalescence during drawing [284,333]. Specifically, this occurs to the elongated second-phase domains owing to the end-to-end contact to which they are subjected during the movements allowed in the molten matrix. As a result, coalescence is obtained and longer fibrils are formed. This mechanism was assessed owing to the introduction of a compatibilizer, promoting the adhesion between the matrix and the dispersed phase but, at the same time, preventing the coalescence between the dispersed droplets due to the formation of a thin film on the particles [20,284,331,335]. Exploiting this approach, Friedrich at al. [56] analyzed the microstructure of PP\PET blends (60:40 wt%) in which 1 wt% of compatibilizer was introduced in substitution to the matrix during compounding. The reduction in the fibril aspect ratio in the presence of the compatibilizer can be clearly appreciated in Figure 28b, in the comparison with the morphology obtained in the drawn PP\PET blend (Figure 28a). This microstructure was attributed to the hindering of the coalescence due to the film of compatibilizer formed on the second-phase surface [56,284,289,336].

**Figure 29:**
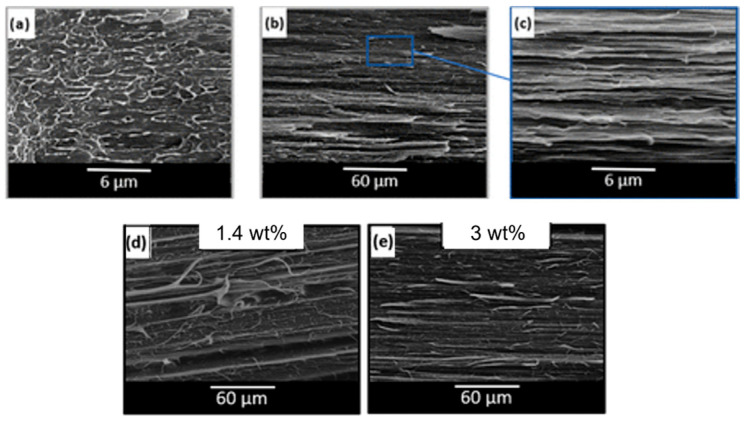
SEM micrographs of LDPE/PP blends without compatibilizers and (**a**) unstretched or (**b**,**c**) drawn. In addition, the morphologies of the fibrillated blends containing (**d**) 1.4 wt% and (**e**) 3 wt% of compatibilizer are reported [288]. Reprinted under CC BY 4.0 license.

In fact, a clear reduction in the aspect ratio of the fibrils after the introduction of the compatibilizer was observed in other studies and attributed to the antagonistic role of the compatibilizer toward the coalescence of the elongated domains [56,287,335,336]. On the other hand, the synergistic effect of the presence of microfibrils and compatibilizer on the mechanical properties was appreciated as well and related to the improved compatibility between the matrix and the dispersed phase [20,81,287,288]. For instance, Rosales et al. [288] studied the effect of different concentrations of compatibilizer (0 wt%, 1.4 wt% and 3 wt%) on the morphology of LDPE/PP blends containing 20 wt% of polypropylene. As can be appreciated in Figure 29b–e, the formation of oriented microfibrils was achieved after the drawing step, and the presence of the compatibilizer in the fibrillated blends (Figure 29d,e) resulted in the improvement in the interaction at the interface of the two materials along with a decrease in the aspect ratio of the fibrils.

The evolution of the microstructure induced variation in the mechanical properties. What emerged was an interplay between the fibril aspect ratio and the compatibilizer content in relation to the increase in the strength at break and modulus [288,337,338]. In fact, a larger surface area was available for the stress transfer in the presence of fibrils instead of droplets, and the compatibilizer enhanced the transfer of the applied stress at the interface between the matrix and the stiffer dispersed phase. However, a limiting content of compatibilizer promoting the mechanical properties was identified, after which the decrease in the tensile properties occurred due to the detrimental reduction in the aspect ratio of the fibrils. For this reason, Kuzmanovic et al. [287] evaluated the possibility of introducing a compatibilizer in PP\PET (80:20 wt%) blends in correspondence to the isotropization step instead of the compounding one, so as to preserve the fibril aspect ratio. What emerged, was the actual increase in the fibril aspect ratio when the compatibilizer was introduced after fibrillation. Nonetheless, the resulting mechanical properties in terms of impact strength, strain at break and yield strength were found to be superior for the blends compatibilized during compounding, despite the reduction in the fibril aspect ratio. This was related to the better interfacial adhesion obtained between the matrix and the second phase when the compatibilizer was added during compounding instead of the isotropization step.

##### The Role of the Viscosity Ratio

Additionally, the viscosity ratio is an important parameter impacting the final morphology because it is related to the formation of fibrils and affects their shape and size [1,20,180,339]. In particular, it was assessed that a low viscosity ratio promotes the formation of high-aspect ratio fibrils, characterized by a narrow diameter distribution. In addition, a uniform distribution of the fibrils in the matrix is promoted. As a consequence, the resulting MFC shows a higher strength and modulus compared to ones with a larger viscosity ratio [20,67,331,332,340,341].

**Figure 30:**
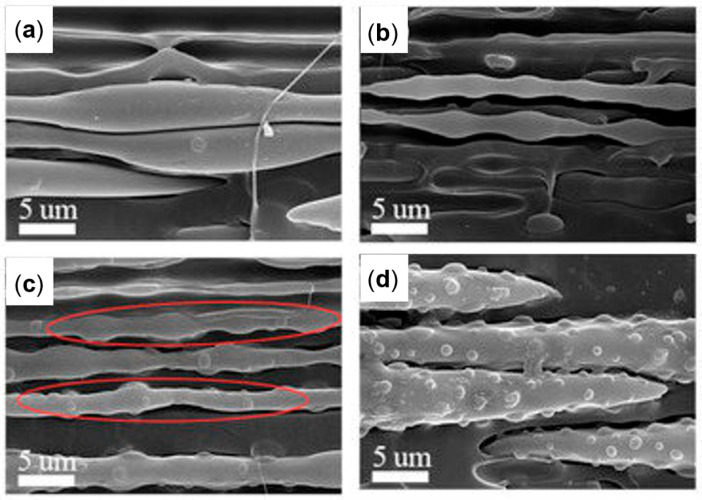
SEM micrographs of PA6\PLA microfibrils in POE matrix with a PA6:PLA viscosity ratio of (**a**) 0.5, (**b**) 2.2, (**c**) 5.3 or (**d**) 14.2 [67]. Reprinted under CC BY 4.0 license.

Interestingly, the viscosity ratio can also be exploited to customize the morphology in blends containing three polymers. This was studied by Shi et al. [67] using polyolefin elastomer (POE)\(PA6\PLA) blends in which different viscosity ratios between PA6 and PLA were exploited. The relative contents of PLA and PA6 were kept constant at a 1:1 ratio, while the ratio between POE and (PA6\PLA) was 75:25. In Figure 30, the SEM micrographs of the materials after the fibrillation step are shown. The morphology corresponding to the lowest viscosity ratio (Figure 30a) is characterized by fibrils with a non-constant diameter, in which coarser and thinner sections follow one another along the axis of the microfibrils. For this reason, the morphology was defined as “gourd-skewers-like”. On the other hand, the uniformity of the diameter of the fibrils increased with the viscosity ratio (Figure 30b,c). In addition, at a larger viscosity ratio (Figure 30c), the formation of bumps on the surface of the fibrils could be appreciated and was particularly evident for the highest viscosity ratio (Figure 30d), for which a “trepang” morphology was obtained.

The morphologies were characterized by different mechanical properties. In all cases, the stress–strain behavior of an elastomeric material was appreciated, although some differences in the values for tensile strength and modulus were observed. In particular, the highest values of both mechanical properties were measured for the viscosity ratio corresponding to the gourd-skewers-like microstructure. Thus, the improvements were related to the enhancement of the surface area characterizing this morphology.

On the other hand, Zhao et al. [342] evidenced the interplay between the viscosity ratio and the second-phase concentration in the formation of the fibrils in PP\PET blends containing between 3 and 30 wt% of the latter. What emerged is that at a low concentration of the second phase, coalescence and fibrillation occurred even at high viscosity ratios, and a good dispersion was achieved. In addition, high aspect ratios could be obtained. On the other hand, at lower viscosity ratios, a greater PET concentration is required in order to obtain coalescence and, thus, high-aspect ratio fibrils. That is to say, the viscosity ratio and second-phase content showed an interplay in determining the limiting concentration for coalescence during drawing [323,342,343].

##### The Role of the Draw Ratio

Another parameter to be taken into account when willing to modify the microstructure in MFCs is the draw ratio. Firstly, with the increase in the DR the progressive deformation of the dispersed-phase domains from spherulitic to ellipsoidal to fibrillar shapes was obtained. Thus, the aspect ratio was improved along with the degree of orientation in the stretching direction. Also, the anisotropy of the matrix was promoted with the enhancement of the orientation of the crystals and the chains of the amorphous phase in accordance with the direction of the drawing applied [1,20,55,69,160,276,297,298,330,344,345,346,347]. However, it is important to note that fibrils are affected by break-up in the case of excessively high DR values. In these cases, the overall anisotropy decreases due to the lowering of the aspect ratio and the orientation of the second-phase domains [297,345,346]. As a result of the transformation of the morphology with the variation in the draw ratio, the tensile properties change with the stretching extent. In fact, a progressive increase in both the strength at break and the Young’s modulus, associated with the decrease in the elongation at break, is obtained with larger DR values owing to the higher aspect ratio obtained and, thus, improved anisotropy. Additionally, for draw ratio values at which the breakup of the fibrils is obtained, a lowering of the strength at break and an increase in the elongation at break can be observed [55,160,276,330,345,346,348].

##### Fibrils as Heterogeneous Nucleation Sites for the Matrix

**Figure 31:**
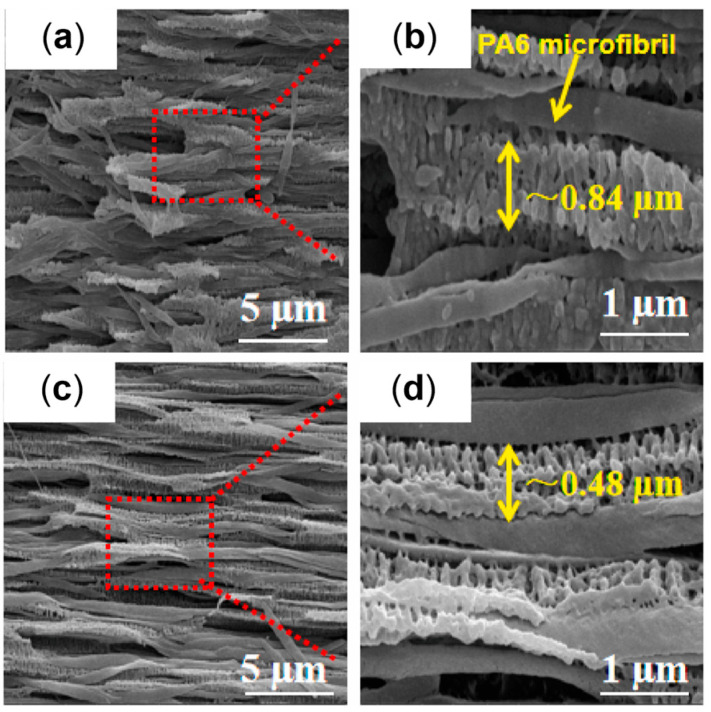
SEM micrographs of 94 wt% PLA/6 wt% PA6 (**a**,**b**) and 88 wt% PLA/12 wt% PA6 (**c**,**d**). [351]. Adapted with permission from Elsevier Science Ltd., 2024.

Lastly, a separate remark has to be made on the effect of the presence of fibrils on the nucleation of the matrix [50,68,297,298,326,327,329,345,349,350,351,352,353,354]. In fact, as already mentioned in the section dedicated to one-step fibrillation, the increase in the aspect ratio of the dispersed phase when fibrils are formed results in a larger surface available for the heterogeneous nucleation of the matrix. Also, the crystallization kinetics of the matrix is promoted [284,295,297,298,326,327,350,353,355]. In addition, the growth direction of the matrix crystals was assessed to be epitaxial to the fibril. As a result, the formation of shish kebabs is promoted and, ultimately, a network-like structure of crystals is created [68,297,327,345,350,351,352,354,355]. On top of that, the heterogeneous nucleation of the crystals in the matrix on the fibrils promotes the interfacial adhesion between the two phases [297,345,352,354]. Additionally, Sun et al. [351] disclosed that the dimension of the epitaxial crystals is influenced by the fibril content. As can be appreciated in Figure 31, the two blends of PLA\PA6 containing 6 wt% or 12 wt% of the latter and processed in the same conditions both show shish-kebab structures. In particular, the PA6 fibrils are the shishes, while the epitaxial PLA crystals are the kebabs (Figure 31b,d). However, the length of the kebabs is negatively affected by the increase in the PA6 content. This is related to the higher density of fibrils formed in the presence of 12 wt% of PA6 compared to 6 wt%, thus representing an obstacle for the growth of the crystals of PLA coordinated by the fibrils themselves.

## 3. Injection Molding

**Figure 32:**
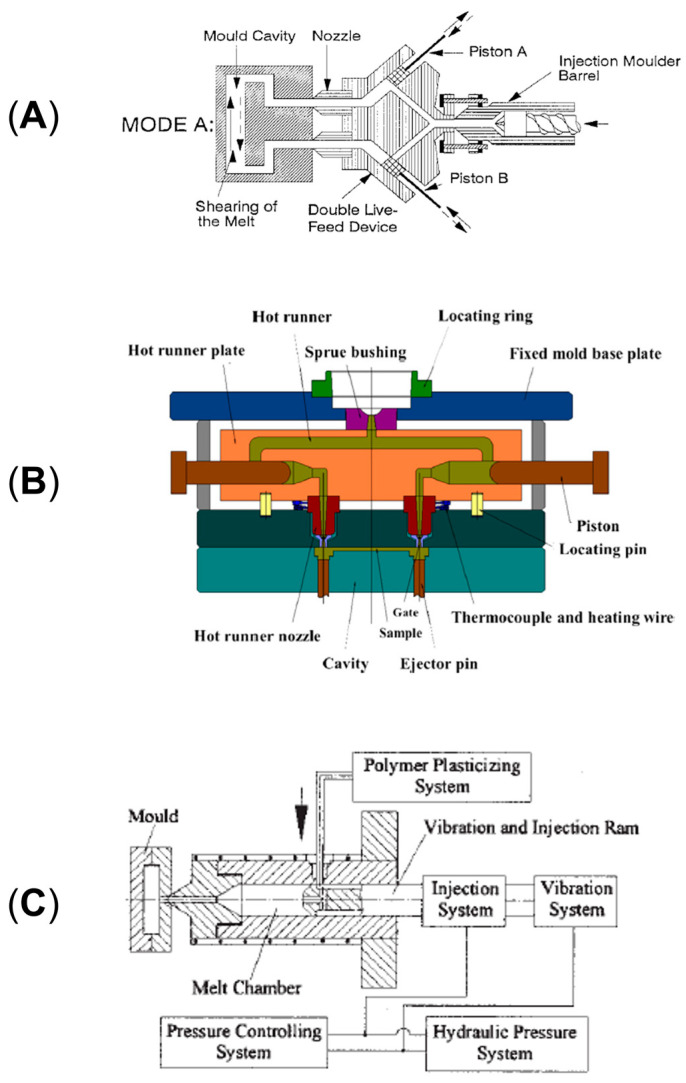
Schematic representations of (**A**) SCORIM [365] (adapted with permission from John Wiley and Sons, 1998), (**B**) OPIM [43] (reproduced with permission from Elsevier Science Ltd., 2015) and (**C**) VIM [401] (adapted with permission from John Wiley and Sons, 2004).

Considering the possible approaches in processing and structuring, one of the most exploited is injection molding (IM) [2,13,14]. In fact, the application of an additional external field which interferes with the relaxation dynamics of the chains in the core layer during the packing and solidification steps also forces the molten polymers to move repeatedly in the mold. As a result, an anisotropic morphology characterized by a high macromolecular orientation is obtained. In order to highlight the primary role of such additional flow in promoting the orientation of the macromolecules and crystallinity, Zhong et al. [2] introduced the term *flow-induced crystallization under pressure* (FICP) to identify all the injection molding techniques that exploit an external field meant to drive morphology structuring. Additionally, these represent an alternative to the introduction of nucleating agents in formulations, which are essential for the microstructuring obtained with conventional IM [13,356,357,358]. In fact, as will be discussed below, different technologies have been developed over the years. For instance, Shear Controlled Orientation in Injection Molding (SCORIM) is characterized by the presence of pistons in the injection barrel [8,14,359,360,361,362,363,364,365,366,367,368,369,370], while in Oscillation Packing Injection Molding (OPIM) such pistons are placed in correspondence to the molding cavity [28,29,30,31,38,42,47,52,371,372,373,374,375,376,377,378,379,380,381,382,383,384,385,386,387,388,389,390,391]. Moreover, a vibration pulse may be applied directly to the material by exploiting the injection screw, as in Vibration-Assisted Injection Molding (VIM) [32,34,35,392,393,394,395,396,397,398,399,400,401]. A schematic representation of the instrumental setup in the three cases is presented in Figure 32 in order to highlight the differences among the techniques. Figure 32A represents SCORIM, Figure 32B OPIM and Figure 32C VIM.

### 3.1. SCORIM

The first achievement reached with this technique is the formation of a trilayer structure owing to the action of the out-of-phase pistons. As a result, the simultaneous presence of a shear and temperature gradient from the skin to the core induces the formation of a third one between the two [13,14,359].

**Figure 33:**
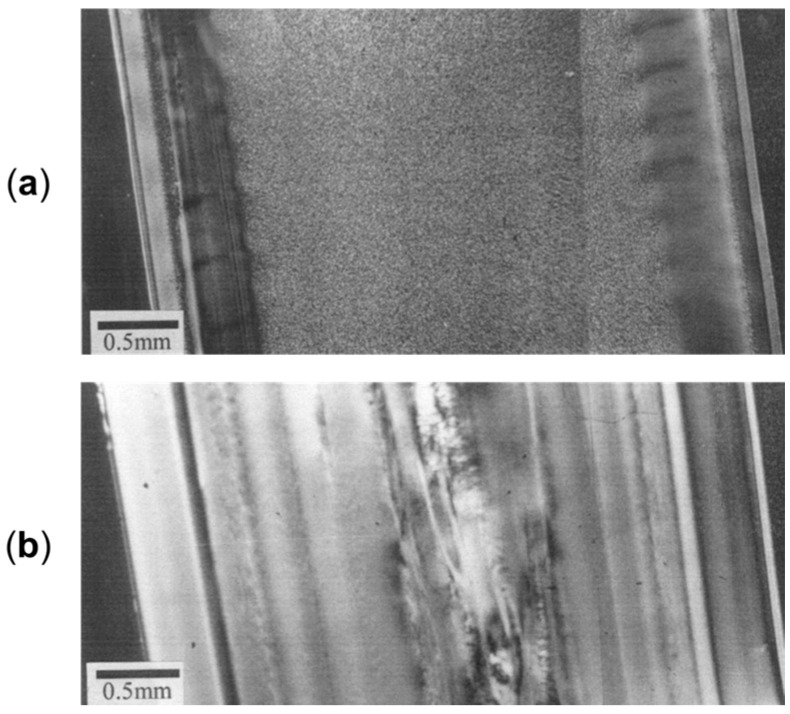
(**a**) CIM and (**b**) SCORIM morphologies of 90 wt% PB/10 wt% PP blends obtained through polarized light microscopy [368]. Adapted with permission from John Wiley and Sons, 2003.

The formation of the trilayer can be clearly appreciated in the study of Kalay et al. [368] on the SCORIM of a blend containing 90 wt% of PB and 10 wt% of PP. In Figure 33, the morphologies of samples processed through conventional injection molding (CIM) (Figure 33a) and SCORIM (Figure 33b) are compared. In the first case, the oriented skin and spherulitic core are clearly distinguished. In addition, a transitory layer between the two is present. On the other hand, in the presence of the external field, the formation of spherulites in the core is suppressed and a thick layered microstructure emerges underneath the skin. As a consequence, the resulting mechanical properties are affected. In particular, an increase in the Young’s modulus from 2083 to 2894 MPa and in the strength at break from 36.8 to 53.1 MPa in the samples processed with SCORIM can be appreciated, notwithstanding a significant decrease in the elongation at break for 195 to 21%.

In addition, Ghosh et al. [369] established the effect of the change in the thermo-mechanical field on the resulting microstructure and properties of poly(L-lactic acid) (PLLA). In particular, the mold temperature was maintained at 30 or 50 °C, while the shearing time was 0, 3, 10 or 15 s. What emerged is that a low mold temperature favors the orientation in the core and that the effect is increased for longer shearing times. On the other hand, the different final orientation did not affect the elastic modulus, and the values were comparable despite the molding temperature and time considered. Additionally, appreciable effects emerged regarding the breaking properties. In particular, the samples produced with SCORIM presented greater strength and elongation at break than the ones obtained with CIM. Additionally, in the case of a molding temperature of 50 °C, both values increased when a longer shearing time was applied.

Such improvement in the mechanical properties of the SCORIM samples was observed also by Kalay et al. [366]. In fact, in their study on PP and aliphatic polyketone (PK), they confirmed the enhancement of the maximum stress and elongation at break for both the materials. However, in opposition to the negligible effect of the transformation process on the Young’s modulus reported by Ghosh et al. [369], in the work of Kalay et al. [366] the positive impact of SCORIM on this property and its enhancement was related to the improved macromolecular orientation and final crystallinity resulting from the SCORIM treatment. In fact, this was also reported in a previous study, in which the increase in the elastic modulus in PP samples was related to the formation of a γ phase instead of the expected β one, owing to the increased macromolecular orientation promoted by SCORIM [361].

**Figure 34:**
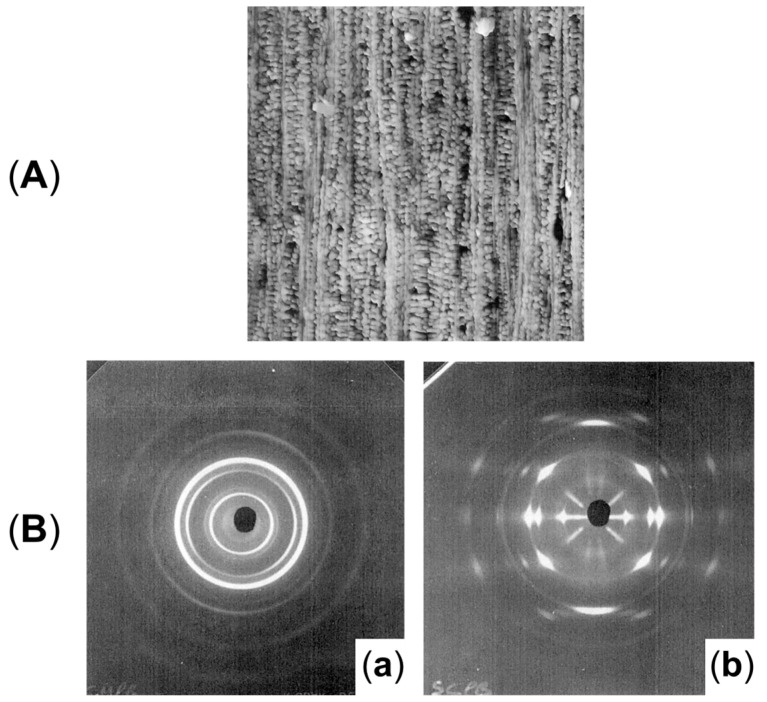
(**A**) AFM image of the SCORIM sample (reported area of 5 × 5 μm and 200 nm as z-scale). (**B**) X-ray patterns of (**a**) CIM and (**b**) SCORIM PB. [8]. Reprinted with permission from John Wiley and Sons, 2003.

Additionally, the structuring ability of the technique is extensively confirmed by the formation of shish kebabs in PB, HDPE and PP [8,360,362,363,364,365,367,370]. For instance, Kalay et al. [8,367] observed such morphologies in samples processed with SCORIM (Figure 34A) but not in ones obtained with CIM, as confirmed by X-ray Debye patterns (Figure 34B, inserts a and b).

On the other hand, the formation of shish kebabs in the SCORIM samples not only positively affected the tensile properties [8,360,362,364,365,367,370] but also the wear resistance [363]. The tribological performances of a 95 wt% HDPE\5 wt% UHMWPE blend was studied by Zhang et al. [363]. What emerged from the comparison of the CIM and SCORIM samples was a different wearing mechanism in the two cases. In particular, micro-fatigue was observed in the former, while mild abrasive wear was appreciated in the latter. Such different tribological behaviors were related to the microstructures resulting from the two techniques. Specifically, the increased orientation reached in the SCORIM samples resulted in a decrease in micro-voids and micro-cracks. Additionally, the reduction in crack development was also related to the formation of shish kebabs and in situ microfibrils observed in the samples.

### 3.2. OPIM

This technique is a variation of SCORIM and may also be referred to as Oscillation Shear Injection Molding (OSIM) or Dynamic Packing Injection Molding (DPIM) [2,13,14]. Also, in this case, the formation of a trilayer structure and the tuning of phase separations in blends can be achieved [377,378,379,380,381,382,383,386,402,403,404]. Additionally, this process is of particular interest for the production of reinforced materials containing shish-kebab structures [28,29,30,31,36,38,42,43,44,46,47,49,52,372,373,374,375,376,384,385,387,388,390,391,405], specifically, deeper near the core, where usually a less-ordered morphology is reached [13,46,47,385,390]. In fact, Zhong et al. [2] highlighted the flexibility of the technique for the production of shish kebabs, despite the macromolecular characteristics of the polymer considered in terms of chain rigidity and length. However, the importance of the flow duration and oscillation frequency in the determination of the final microstructure emerged.

For instance, Sang et al. [28] studied the effect of such parameters on the morphology of PLA bars. The samples were obtained at two oscillation frequencies, namely, 0.5 and 2 Hz, and five oscillation times: 6, 18, 30, 90 and 120 s. For the sample obtained through CIM, a low crystallinity content and poor orientation emerged throughout the section, while the ones obtained with OPIM were characterized by the presence of a structured layer. Additionally, the thickness toward the core increased when longer exposure times or oscillation frequencies were exploited.

**Figure 35:**
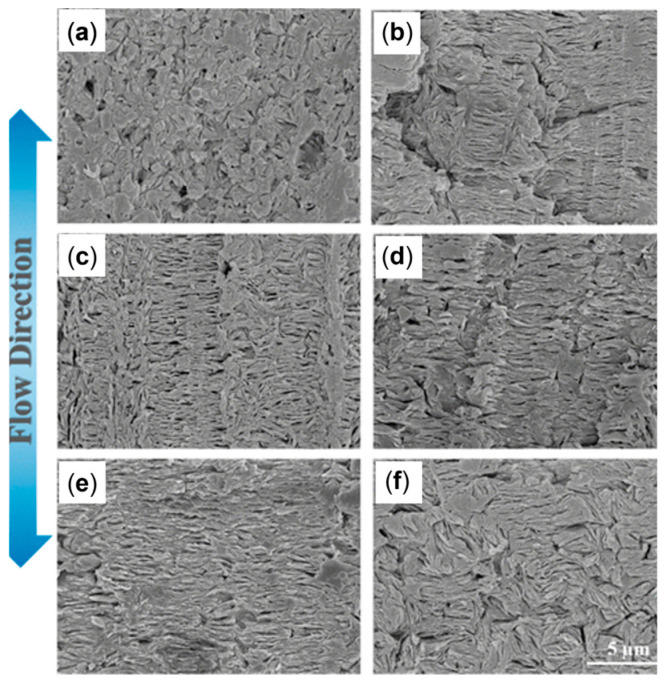
SEM micrographs at 800 μm from the surface of PLA processed for different oscillation times and at different frequencies: (**a**) CIM, (**b**) 6 s at 0.5 Hz, (**c**) 18 s at 0.5 Hz, (**d**) 30 s at 0.5 Hz, (**e**) 90 s at 0.5 Hz and (**f**) 120 s at 2 Hz. [28]. Reproduced with permission from the American Chemical Society, 2017.

Such observations can be clearly appreciated from the SEM micrographs at 800 μm from the surfaces of the samples processed in the different conditions shown in Figure 35. In fact, it emerges that the spherulitic structure obtained with CIM (Figure 35a) progressively evolves toward an oriented-lamellae conformation in the direction perpendicular to the flow with the increase in the oscillation time at a constant oscillation frequency (Figure 35b–e). In addition, the formation of shish-kebab structures aligned in the direction parallel to the flow was obtained in the samples processed at 0.5 Hz for 6 and 18 s (Figure 35b,c), while for the greatest oscillation frequency and time (Figure 35f), distorted spherulites were observed.

Additionally, the changes in the microstructure described so far impacted the resulting Heat Deflection Temperature (HDT), and a major role for orientation compared to thickness was assessed [28]. In fact, the HDT increased from 55.7 °C for the CIM sample up to 76.5 °C when the oscillation was applied for 18 s and the crystalline thickness corresponded to 1200 μm. Also, the value remained almost constant for longer exposure times. However, when the oscillation frequency increased from 0.5 to 2 Hz, the HDT improved from 78.9 °C to 96.6 °C, even though the same oriented-layer thickness of 2000 μm characterized the two.

The above-mentioned improvement in the final characteristics of a single polymer through processing and structuring is also known as “self-reinforcement”, and the effectiveness of OPIM in reaching such results was reported also for PP and PE matrices [29,30,31,373,374]. Additionally, other studies were in accord regarding the impact of macromolecular structuring and the formation of shish kebabs on the mechanical properties. For instance, Xie et al. [38] produced a 90 wt% PBS\10 wt% PLA blend with OPIM, achieving an increase in the tensile strength and modulus from 38 to 49 MPa and from 525 to 634 MPa, respectively, when the material was processed with OPIM instead of CIM. These data were interpreted considering the formation of interlayered shish-kebab structures in the direction parallel to the flow for the blend obtained in the presence of the external field. In fact, the variations in the tensile properties can be explained considering two different effects: (i) the larger stress that the shishes can withstand compared to isotropic crystals and (ii) the intermeshing of the kebabs that prevents macromolecules from slipping. On top of that, the amorphous phase between the lamellae has a role in transferring the applied load to the stiffer shish-kebab structures.

All the above is in agreement with the results of the study of Xu et al. [42] on a 95 wt% PLA\5 wt% poly(ethylene glycol) (PEG) blend. However, even if the improvement in the mechanical properties in the OPIM samples was appreciated, the decrease in the ductility in the treated samples was not as dramatic as in the previous cases. In particular, the deformation at break was 18.7% for the CIM sample and 11.3% for the OPIM one. In addition, the improvement in the impact strength from 5.0 to 10.6 KJ/m^2^ was related to the presence of the bamboo-like hierarchical morphology in the samples processed in the presence of the external field. This peculiar skin–core morphology involved a high density of shish-kebab structures characterizing the crystalline skin layer and an isotropic spherulitic morphology in the core. In such situations, the hierarchical anisotropic skin represents a sort of strong shell for the softer internal structure, which, by contrast, is particularly prone to absorbing a greater amount of impact energy when compared to an oriented morphology [42,405].

**Figure 36:**
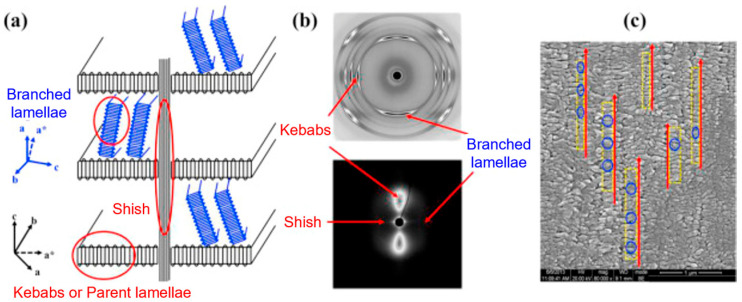
(**a**) Schematic representation of the hierarchical shish-kebab structure, in which the shish, primary and branched kebab lamellae are distinguished. (**b**) WAXD and SAXS patterns of the samples of isotactic PP produced with OPIM. (**c**) SEM micrograph of the sample, in which the red arrows indicate the shishes, the yellow rectangles represent the external perimeters of the kebabs and the blue circles highlight the secondary lamellae [36]. Adapted with permission from Elsevier Science Ltd., 2015.

Additionally, the shish kebabs themselves can be further structured, thus providing additional levels in the hierarchical arrangement. This is the case with the microstructure observed by Chen et al. [36] in isotactic PP produced via OPIM. In fact, as emerges from Figure 36b, the WAXD and SAXS analyses highlight the formation of an additional level of lamellae, named “branched lamellae”, which, as can be appreciated from the SEM micrographs (Figure 36c), are stacked between the primary ones constituting the kebabs. Such additional structures correspond to the branching of the kebabs. A schematic representation of the hierarchical morphology is shown in Figure 36a. In addition, in the study, the formation mechanism of these branched lamellae was suggested. Specifically, it was claimed that the primary kebab lamellae act as nucleation sites for the secondary ones; thus, the latter are oriented according to the angle of the α-phase monoclinic unit cells (99°80′) considering the face of the kebabs.

Such a morphology also had an important impact on the mechanical properties. In fact, a homogeneous deformation without necking was observed during the tensile tests, and this behavior was related to the presence of the secondary branches [36]. In particular, at the beginning of the deformation, the separation of the primary lamellae induced the rotation of the secondary ones in the direction parallel to the drawing. Thus, in the early stages, the reduction in the lateral dimension was prevented by the change in the internal alignment. Then, with the increase in the axial deformation, the destruction and reorganization of both the primary and secondary lamellae resulted in the alignment of the macromolecules in the drawing direction and the formation of new oriented crystals. Such a phenomenon is also known as the stress-induced crystal fragmentation and recrystallization process [36,406,407,408,409,410,411].

**Figure 37:**
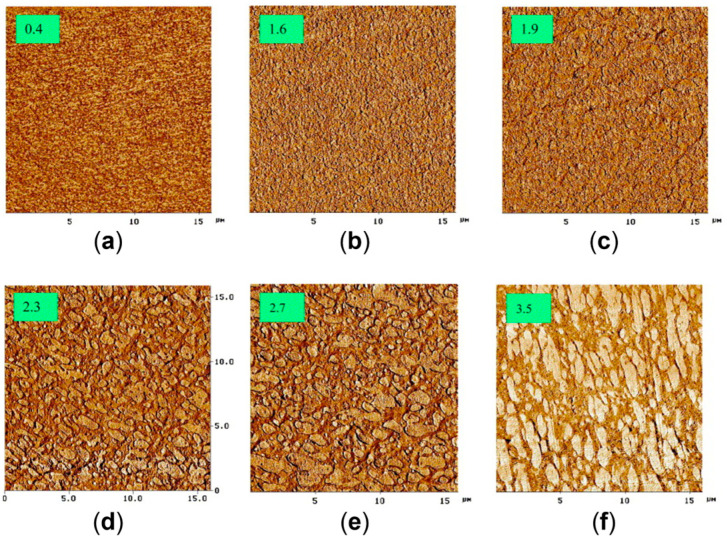
AFM micrographs of OPIM 50 wt% PP/50 wt% LLDPE. The number reported in each photo corresponds to the distance in mm from the skin layer: (**a**) skin layer; (**b**,**c**) sheared layer; (**d**–**f**) core [44]. Adapted with permission from Elsevier Science Ltd, 2004.

Additionally, refinement of the phase separation in polymer blends, together with the formation of shish kebabs, can also be obtained, as reported by Wang et al. [44] for 50 wt% PP\50 wt% LLDPE. In Figure 37, the AFM micrographs of the section of the OPIM sample at different distances from the skin are shown. Firstly, different morphologies depending on the depth were appreciated and related to the punctual combinations of cooling and shear rates. In fact, when both of them are high, a co-continuous phase is obtained (Figure 37a). On the other hand, with their progressive decrease, the formation of an island–sea structure is promoted and refined (Figure 37b–e). It is important to note that the morphology at the core (Figure 37f) was attributed not only to the lower shear rate but also to the increased heat quantity that the material receives due to the active shear flow. 

Additionally, the X-ray analyses highlighted the different chain organizations in the PP and LLDPE domains. In particular, in the first case, oriented shish-kebab structures were distinguished, while in LLDPE two orientations of the lamellar structures were appreciated: in the direction perpendicular to the flow direction and tilted and angled 45–50° from it. Importantly, such different chain organizations were observed regardless of the depth considered. As a result, in the work, a complex hierarchical structure characterized by a macromolecular and a micrometric level was reached owing to the OPIM technique.

**Figure 38:**
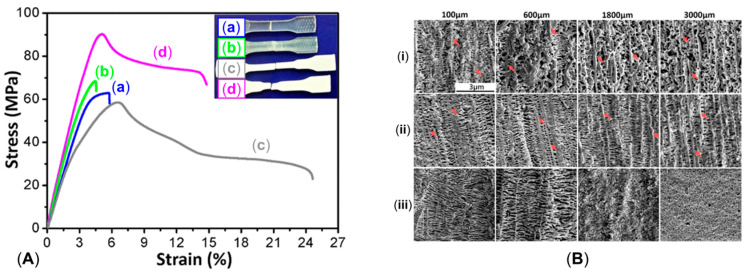
(**A**) Stress–strain curves and corresponding tested samples of (a) CIM PLA, (b) OPIM PLA, (c) CIM 90 wt% PLA/10 wt% PBAT and (d) OPIM 90 wt% PLA/10 wt% PBAT. (**B**) SEM micrographs at different depths of selectively etched (**i**) CIM 90 wt% PLA/10 wt% PBAT, (**ii**) OPIM 90 wt% PLA/10 wt% PBAT and (**iii**) OPIM PLA; the red arrows indicate the positions of PBAT nanofibrils [49]. Adapted with permission from the American Chemical Society, 2017.

In fact, the possibility of reaching a complex hierarchical structure thanks to the OPIM technique has been investigated over the years in order to highlight the important effect on the final properties. For instance, Zhou et al. [49] have proposed a solution to achieve both strength and toughness in PLA. In particular, the exploitation of OPIM was evaluated on both PLA and a 90 wt% PLA\10 wt% PBAT blend. As shown in Figure 38A, OPIM PLA presents an increased strength at break and Young’s modulus and a slightly reduced elongation at break as compared to its CIM counterpart. However, with the introduction of 10 wt% PBAT, the final deformation increases at the expense of the maximum strength when compared to PLA. The further application of OPIM to the blend promoted an increase in the maximum strength from 58.6 to 91.2 MPa and in the elastic modulus from 1649 to 2458 MPa, while lowering the elongation at break from 25.2 to 15.7% compared to CIM 90 wt% PLA\10 wt% PBAT.

This overall enhancement of the tensile properties was explained by the microstructures in the different cases. In Figure 38B, it can be observed that the formation of PBAT nanofibrils was obtained in both CIM (insert i) and OPIM (insert ii) blends and that these structures are present at different depths from the skin. However, the diameter of the fibrils depends on the production technique and decreases from about 300 nm in the CIM samples to less than 150 nm in the OPIM ones owing to the deformation of the second phase promoted by the intense shear flow present. Additionally, PLA takes an active part in the definition of the hierarchical morphology. In fact, aligned lamellae of matrix oriented perpendicularly to the PBAT fibrils embed the second phase in both CIM and OPIM blends (Figure 38A, i and ii). On the other hand, as can be appreciated in Figure 38B (insert ii), the simultaneous presence of the second phase and the external field has a synergistic effect on the refinement of the microstructure, with the formation of structured shish kebabs throughout the thickness of the sample. In particular, the wrapped nanofibrils of PBAT constitute the core of the shish and coordinate the shear-flow-induced stretching of the PLA macromolecules forming a layer of shish surrounding the external surface of the PBAT shish itself. This phenomenon is allowed owing to the low interfacial tension and large specific surface between PLA and the second phase. Further, the low interfacial tension along with the partial miscibility between the two polymers plays a role in the stability of the matrix shish layer. In fact, the slow relaxation of the PBAT nanofibrils suppresses the collapse of the PLA macromolecules, preserving their orientation once the shear ceases. On top of that, the determinant role of PBAT in the refinement of the morphology is confirmed based on the comparison of the morphology of the blend (Figure 38B, insert ii) and that of pristine PLA (Figure 38B, insert iii) processed with OPIM. In fact, a poorer alignment of the PLA shish kebabs can be appreciated in the second case, and the anisotropy is limited to the skin layers, where a shorter time for relaxation is provided for the macromolecules prior to solidification.

Keeping this in mind, the mechanical behavior in Figure 38A can be understood. In fact, the increase in the tensile strength is related to the enhancement of the shish-kebab density and orientation, while the presence of ductile nanofibrils ensures the retainment of a good toughening by facilitating the stretching of the PLA chains without affecting the overall strength.

In fact, the importance of exploiting a blend in order to promote morphological stability and refine shish kebabs has been confirmed in several studies [43,46,52,375,376,384,385,387,390,391]. Additionally, the importance of the second-polymer characteristics on the final microstructure has been investigated. For instance, Liang et al. [46,390] highlighted the positive impact of the introduction of a low-MW HDPE instead of a high-MW HDPE in an LLDPE matrix. In fact, although in all cases the formation of shish-kebab structures was observed, the higher degree of shish orientation and the more compact kebab lamellae formed in LLDPE were related to the greater chain mobility of the low-MW HDPE along with the change in the miscibility between the matrix and the second phase, which ultimately were beneficial for the overall anisotropy.

**Figure 39:**
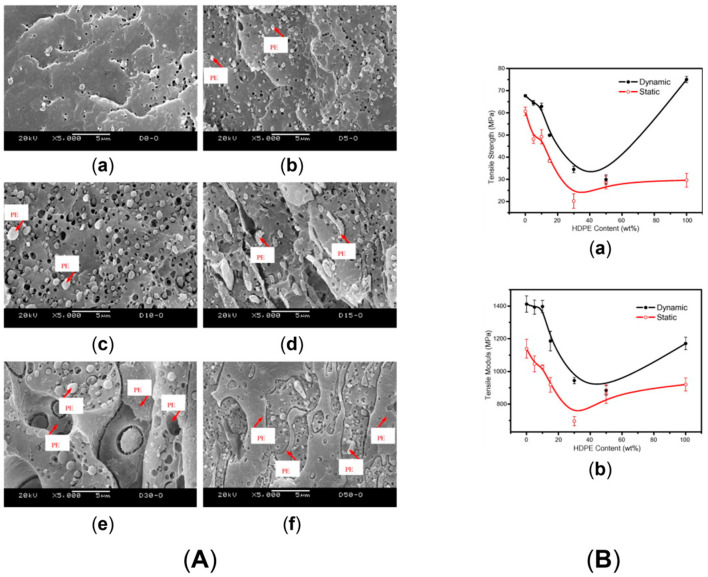
(**A**) SEM micrographs of the shear zone sections of OPIM (**a**) POM, (**b**) 95 wt% POM\5 wt% HDPE, (**c**) 90 wt% POM\10 wt% HDPE, (**d**) 85 wt% POM\15 wt% HDPE, (**e**) 70 wt% POM\30 wt% HDPE and (**f**) 50 wt% POM\50 wt% HDPE. (**B**) Tensile (**a**) strength and (**b**) modulus of the corresponding blends obtain with CIM (static) and OPIM (dynamic) [386]. Adapted with permission from Elsevier Science Ltd., 2009.

Lastly, the formation of shish-kebab structures is not always observed in samples produced with OPIM, but, nevertheless, improvement in the mechanical and impact properties can be observed when they are compared to CIM samples [377,378,379,380,381,382,383,386,402,403,404]. However, the application of an external field does not prevent a decrease in the tensile features with the increase in the minor-phase content [382,383,386], as in the case of the polyoxymethylene (POM)\HDPE blends studied by Su et al. [386]. The content of HDPE was 5, 10, 15, 30 or 50 wt%. In Figure 39A, SEM micrographs of the shear layers of the OPIM samples are shown. In all cases, phase separation can be appreciated. Additionally, the progressive coalescence and formation of a co-continuous phase of HDPE can be observed with its increasing content (from 5 wt% in Figure 39A, insert b, to 50 wt% in Figure 39A, insert f). In particular, the minimum concentration for a co-continuous path was identified as 15 wt% in the work. In addition, the sub-inclusion of one polymer in the co-continuous phase of the other was observed.

In the study, the coalescence phenomena were attributed to the presence of the external field which, however, was not able to further promote the orientation of the immiscible polymers’ domains due to their mutual hindering effects. For this reason, even if an increase in the tensile strength and modulus was observed in comparison with the CIM samples (Figure 39B), the performance decreased with the increase in the HDPE content.

### 3.3. VIM

This technique is particularly effective in the self-reinforcement of a single polymer through the layering and formation of shish-kebab structures [2,13,14,32,34,35,389,393,394,395,396,397,399,400,401,412].

**Figure 40:**
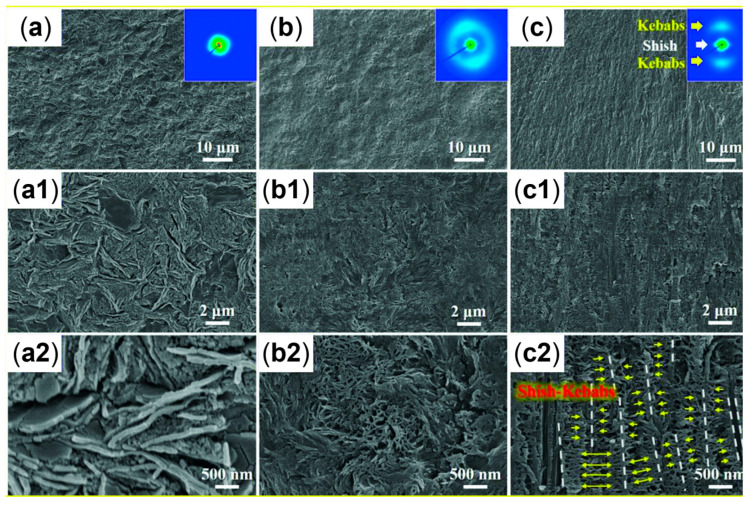
SEM micrographs of samples obtained with a vibration frequency of (**a**) 0 s^−1^, (**b**) 12 s^−1^ or (**c**) 30 s^−1^. Images (**a1**,**a2**,**b1**,**b2**,**c1**,**c2**) are magnifications of (**a**–**c**). Also, the resulting SAXS is reported. The flow is in the vertical direction, and the white lines represent the shishes [34]. Adapted with permission from John Wiley and Sons, 2018.

An interesting example of the potentiality of the application of vibration is represented by the study of Yang et al. [34] on PVDF, where the impact of different vibration frequencies was investigated. In Figure 40, the resulting morphologies of samples processed with a frequency of 0, 12 or 30 s^−1^ are shown. Firstly, the progressive increase in anisotropy with the enhancement of the vibration frequency can be observed. In fact, while the sample processed through CIM was isotropic, an improvement in the overall orientation was obtained at 12 s^−1^; besides, shish-kebab structures oriented along the flow direction were present in the sample processed at 30 s^−1^.

On the other hand, considering the polymorphism of PVDF, it emerged that with the enhancement of the vibration frequency, the formation of the less stable β and γ phases was reached. In particular, in the CIM sample only the α phase was present, while with the frequency of 12 s^−1^ the β phase could also be distinguished. Lastly, in the sample processed at 30 s^−1^ the simultaneous presence of α, β and γ was observed.

**Figure 41:**
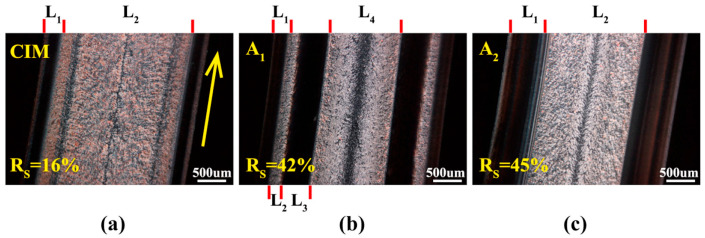
PLM images of samples obtained with (**a**) CIM, (**b**) OPIM with a long static interval and (**c**) OPIM with a short static interval, where R_s_ represents the ratio between the shear layer and the overall section thickness. The arrow represents the flow direction [389]. Reproduced with permission from Elsevier Science Ltd., 2017.

In addition, an alternated morphology made of shear layers and spherulite layers may be achieved with the purpose of altering the crack deflection mechanism and improving the impact strength, as in the case of the works of Hou et al. [32,389] on PP. In Figure 41, the Polarized Light Microscopies (PLMs) of the samples produced in different conditions are shown and the impact of the VIM technique can be clearly appreciated. In particular, Figure 41a shows a CIM sample, which is characterized by an evident skin–core structure and a thin shear layer. Considering the skin and shear layer as a whole (R_s_), it can be quantified as 16% of the overall section; thus, most of it is constituted by the spherulitic core region. On the other hand, the samples processed via VIM show a tunable morphology. Specifically, the layering of spherulitic and shear layers is obtained owing to the alternation of static and vibration conditions in the cavity. Additionally, by appropriately deciding the duration of the external field application, the thickness of the different layers can be defined. This can be clearly appreciated when comparing Figure 41b,c. In fact, in the second case, no spherulites in the core were present because a lower static period was allowed for the cooling of the material in the center. As a result, two different multilayer structures characterized by the same R_s_ values were obtained. On top of that, an additional complexity level was highlighted because shish-kebab structures parallel to the shear flow were observed in the shear layer. Also, their density and radial diameter were related to the cooling rate during the solidification. In fact, a lower density of shishes characterized by larger kebabs was obtained in correspondence to the mold walls, while a greater density of thinner shish kebabs was reached in the internal shear layer. Thus, a hierarchical tunable microstructure was produced.

As a consequence, different final properties were achieved. In particular, the plastic deformation observed in the spherulitic layers of VIM samples after the impact tests was greater than that shown by the samples processed through CIM [389]. Additionally, the rough fracture surface of the shear layers attested to the role of the shish kebabs in facing the impact loading. In addition, the plastic deformation in the shear layer was greater than that observed in the spherulitic one. A possible explanation of this phenomenon was provided by the energy transferred during the impact from the shish kebab to the surrounding area, resulting at the same time in improved energy absorption and more extended plastic deformation. Taking this into consideration, the impact behaviors of the two OPIM samples (Figure 41b,c) differ due to the different distributions of the oriented and isotropic layers and the greater resistance compared to the ones shown in Figure 41b, due to the higher efficiency in crack deflection [389,413]. However, the tuning of the microstructure has an almost negligible impact on other mechanical properties. In fact, the tensile strength is mainly determined by the overall crystallinity instead of the layered structure; thus, the resulting performances of samples characterized by a comparable R_s_ are similar, despite the layering [32,389].

**Figure 42:**
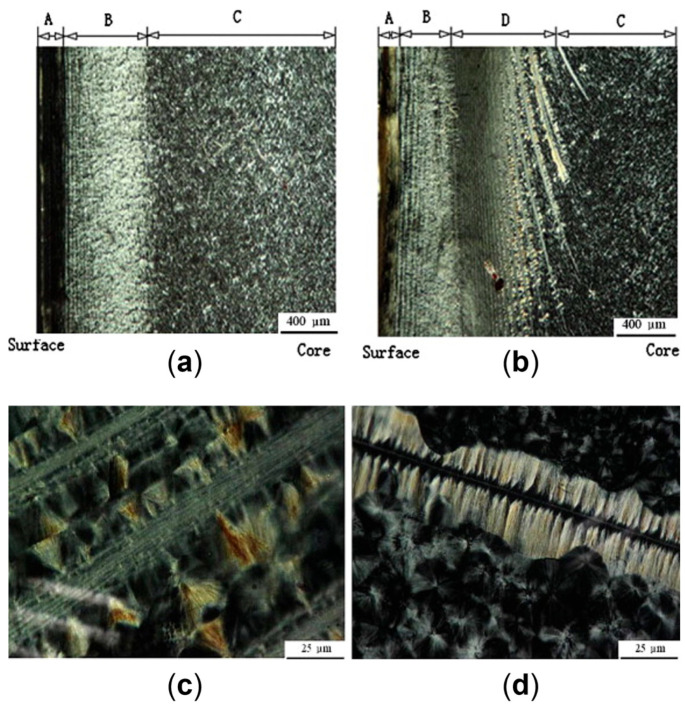
PLM images of cross-sections of the samples prepared with (**a**) CIM and (**b**) VIM and magnifications of the (**c**) multi-fibrillar and (**d**) single-fibril cores. A indicates the skin layer, B the transition layer, C the core and D the shish-kebab-like cylindrulite region [35]. Adapted with permission from Elsevier Science Ltd., 2011.

On the other hand, even more complex morphologies were reported to be obtained with VIM, such as the shish-kebab-like cylindrulite structures in PP [35,399]. This was observed by Zhou et al. [35] in samples processed at a frequency of 1.10 Hz. The expected layered morphology was appreciated in the CIM sample (Figure 42a) with skin (A), core (C) and transition layers (B) characterized by the presence of shish kebabs, spherulitic structures and an anisotropic transition microstructure, respectively. In addition, an additional fourth layer characterized by the presence of shish-kebab-like cylindrulites (*cylindrulite* hereafter) was distinguished in the VIM sample (Figure 42b,d). As mentioned above, the morphology of the cylindrulite depends on the distance from the surface. In fact, while the cylindrulites next to the core are smaller and are characterized by a single-fibril shish, in those closer to the surface the shishes are multi-fibrillar structures (Figure 42c). The observed difference was explained by the authors in terms of the progressively lower shear affecting the material during solidification from the skin to the core. In fact, the cross-sectional area of the gate progressively solidifies during the application of the external vibration, resulting in a corresponding decrease in the shear stress from the skin layer to the core. As a result, more vibrations are transferred in the first case than in the second and the orienting effect will involve a larger number of chains closer to the surface, with a smaller number next to the core. For this reason, the shishes closer to the skin are multi-fibrillar, while the others are made of a single fibril (Figure 42d). Additionally, in both cases the oriented macromolecules corresponding to the future shish structures act as nucleating sites for the chains forming the epitaxially oriented lamellae.

## Data Availability

Not applicable.
